# A hybrid method for water stress evaluation of rice with the radiative transfer model and multidimensional imaging

**DOI:** 10.1016/j.plaphe.2025.100016

**Published:** 2025-02-28

**Authors:** Yufan Zhang, Xiuliang Jin, Liangsheng Shi, Yu Wang, Han Qiao, Yuanyuan Zha

**Affiliations:** a State Key Laboratory of Water Resources Engineering and Management, Wuhan University, Wuhan, 430072, Hubei, China; b Institute of Crop Sciences, Chinese Academy of Agricultural Sciences/Key Laboratory of Crop Physiology and Ecology, Ministry of Agriculture, Beijing, 100081, China

**Keywords:** Water stress, Hyperspectral, Computer vision, Machine learning, Radiative transfer model

## Abstract

Water stress is a crucial environmental factor that impacts the growth and yield of rice. Complex field microclimates and fluctuating water conditions pose a considerable challenge in accurately evaluating water stress. Measurement of a particular crop trait is not sufficient for accurate evaluation of the effects of complex water stress. Four comprehensive indicators were introduced in this research, including canopy chlorophyll content (CCC) and canopy equivalent water (CEW). The response of the canopy-specific traits to different types of water stress was identified through individual plant experiments. A hybrid method integrating the PROSAIL radiative transfer model and multidimensional imaging data to retrieve these traits. The synthetic dataset generated by PROSAIL was utilized as prior knowledge for developing a pre-trained machine learning model. Subsequently, reflectance separated from hyperspectral images and phenotypic indicators extracted from front-view images were innovatively united to retrieve water stress-related traits. The results demonstrated that the hybrid method exhibited improved stability and accuracy of CCC (R ​= ​0.7920, RMSE ​= ​24.971 ​μg ​cm^−2^) and CEW (R ​= ​0.8250, RMSE ​= ​0.0075 ​cm) compared to both data-driven and physical inversion modeling methods. Overall, a robust and accurate method is proposed for assessing water stress in rice using a combination of radiative transfer modeling and multidimensional image-based data.

## 1 Introduction

Climate change-induced droughts are becoming increasingly frequent, posing a significant threat to global rice yields [1]. The application and promotion of the deficit-regulating irrigation strategy have achieved water conservation and yield improvement [2]. The intermittent wet and dry pattern of water management complicates the spatial and temporal variation of soil moisture in rice fields.

Measurement of plant physiological traits serves as the primary approach for assessing crop water stress. Key indicators, including organ water content, dry matter accumulation, canopy water vapor flux, and light use efficiency are pivotal components in the evaluation process [3,4]. Leaf water content is frequently utilized as a moisture index due to its intuitive and accurate nature [5]. Furthermore, a comprehensive understanding of crop drought conditions can be obtained through the canopy water content index, which accounts for all leaf water, and the plant water content, which encompasses aboveground organ water [6]. While organ water content indexes provide valuable insights into plant water physiology, there is a risk of overgeneralization in the evaluation of water stress [7]. The risk stems from the fact that these indexes typically provide an aggregate or average measurement of water content across various plant parts, thereby potentially obscuring the inherent variability in water status among individual organs or tissues. Given the close link between water movement and energy exchange processes, canopy temperature gaps, relative humidity, and stomatal conductance can serve as indicators of water stress impacts [8,9]. Conversely, the canopy structure of rice exhibits morphological differences under varying drought conditions [10], resulting from multiple intrinsic hormonal modulations. These structural characteristics, quantified through shape descriptions such as leaf area index (LAI), leaf inclination angle (LIA), and leaf rolling, allow for the perception of water stress effects at various scales [11,12].

Ground-based spectral measurements provide a highly precise and noninvasive means of monitoring crop water status. Specifically, the reflectance of certain wavelengths is sensitive to changes in plant water content [[13], [14], [15]]. Vegetation indices, calculated through a combination of multi-band spectral data, enable the discernment of growth differences attributable to water stress. Spectral vegetation indices, represented by NDVI (normalized difference vegetation index), have proven to be particularly valuable for assessing and evaluating water stress, inverting water physiology, and reflecting growth status [16,17]. The advent of various vegetation indices has broadened the options for retrieving plant traits, and on the other hand, increased the complexity of spectral evaluation methodologies [18,19]. To address the problem, the application of machine learning for direct information from reflectance data has emerged as a more efficient approach [20,21]. Multi-source data fusion enhances the comprehensiveness of information available for depicting vegetation traits. Beyond spectral reflectance, the incorporation of additional accessible features into drought identification and evaluation not only enriches the data dimensions but also signifies a deeper understanding of drought impacts. For instance, the inclusion of LAI improves the accuracy of predicting soil moisture content [22], while a priori knowledge of canopy structure can optimize the estimation of leaf pigments [23]. Employing data-driven methods facilitates the selection of appropriate vegetation indices for water stress evaluation from the extensive array of available approaches.

The radiative transfer process is among the heavily influenced processes correlated with soil and plant water change. It encompasses the interaction of canopy space, soil surface, and incoming sunlight, involving light interception, reflection, transmission, and refraction [24]. The PROSAIL model is one of the prominent physical models developed for parameterizing the radiative transfer links and describing light travel. It combines the PROSPECT leaf optical properties model and the SAIL canopy bidirectional reflectance model [25,26]. Using radiative transfer models to retrieve physiological traits offers an indirect approach to identifying and evaluating the effects of crop water stress. However, establishing the correlation between model parameters and drought effects is crucial [27]. Simultaneously, an appropriate parameter search space and a fast numerical iteration method are necessary for a robust retrieval approach [28,29]. Furthermore, model parameters should be adapted and optimally fitted to better approximate real-world conditions, moving beyond empirical definitions. Incorporating distributed leaf inclination angle parameters enhances the accuracy of PROSAIL model forward simulations [30,31]. Precise measurements, detailed priori information, and a suitable parameter structure together contribute to the accuracy of the retrieval model.

Data-driven methods demonstrate rapid convergence but require substantial measured data. Physical models provide a parameter tuning space with mechanistic implications but lack specificity for particular problems. Hybrid approaches provide an opportunity for these methods to complement each other [29]. The mathematical-physical method of coupling the PROSAIL model and variational heteroskedasticity Gaussian process regression (VHGPR) enables effective estimation of soil moisture and the creation of regional moisture maps by incorporating the actual distribution of LAI [22] Notably accurate crop traits evaluations have been achieved through the development of a radiative transfer process-guided machine learning model (PGML). The workflow involves pre-training the machine learning model using extensive virtual data generated from the radiative transfer model, followed by fine-tuning it with a limited amount of measured information [32,33]. A hybrid machine learning approach using the PROSAIL model has been widely used for crop radiative transfer simulation and inversion of related traits. However, for the complex water conditions in rice fields, it is difficult for individual model parameters to accurately capture the impacts of drought, thus more phenotypic responses need to be considered. Meanwhile, high-throughput and low-cost crop monitoring is the key to realizing model coupling, and how to obtain more comprehensive information from more simplified observation means the current development direction of agricultural phenotyping research [34,35].

In the practice of alternating wet and dry irrigation patterns, contemporary research has primarily concentrated on elucidating the physiological regulation of rice and alterations in microbial dynamics and solute transport within the soil [4,36]. These microscopic mechanisms are instrumental in fostering a comprehensive understanding of drought resistance and rehydration compensation effects in rice crops. However, a significant challenge persists in quantifying these micro-physiological transformations based solely on macroscopic phenotypic observations. To overcome the hurdle in rice water stress evaluation, we investigated the indirect impacts of fluctuating water conditions on the radiative transfer process, revealing that the interplay of related traits within this process holds promise for quantifying both long- and short-term drought effects. The study aims to delve into the application of machine learning, specifically through the coupled PROSAIL model, to analyze rice responses to alternating drought-rehydration scenarios. To this end, a two-year experiment involving water-deficit treatments of rice was conducted. The primary objectives were (1) To analyze the response of rice physiological indicators related to radiative transfer under water stress, (2) To refine the PROSAIL model parameters by integrating phenotypic features derived from front-view images and other specialized measurements, imparting priori knowledge, (3) To develop a hybrid retrieval method that synergizes data-driven and process-guided models, leveraging multidimensional imaging data, particularly hyperspectral data, to extract water stress traits and enhance the accuracy and reliability in drought identification and evaluation.

## 2 Materials and methods

### 2.1 Experimental site and the water treatments

In 2021 and 2022, controlled drought experiments were conducted on rice plants at the Irrigation and Drainage Experimental Site of State Key Laboratory of Water Resources Engineering and Management, Wuhan University (114°37′E, 30°54′N). Rice plants were cultivated in pots with a diameter of 20 ​cm and a depth of 25.5 ​cm, featuring impermeable bottoms. Each pot was initially filled with 6.4 ​kg of air-dried soil, maintaining a bulk density of 1.2 ​g ​cm^−3^. The parameters of soil for experimental cultivation were provided in Supplementary Table 1. Throughout the two-year experimental period, we meticulously controlled the level of water stress by adjusting the soil moisture content (SMC). This was accomplished by regulating the water supply to the plants, thereby inducing specific levels of water deficit. To prevent rainwater interference, the potted rice plants were positioned under a motorized roof. Utilizing soil moisture retention curves obtained from preliminary experiments, we were able to accurately estimate the soil volumetric moisture content by weighing the potted plants and calculating the necessary amount of water for rehydration. Throughout the two-year experiment, a total of 272 pots of rice samples (169 pots in 2021 and 103 pots in 2022) were set up. The experimental periods were as follows: in 2021, the experiments commenced on June 1st and the rice was harvested on October 13th; in 2022, the experiments were initiated on June 3rd, and the rice was harvested on October 7th.

**Fig. 1:**
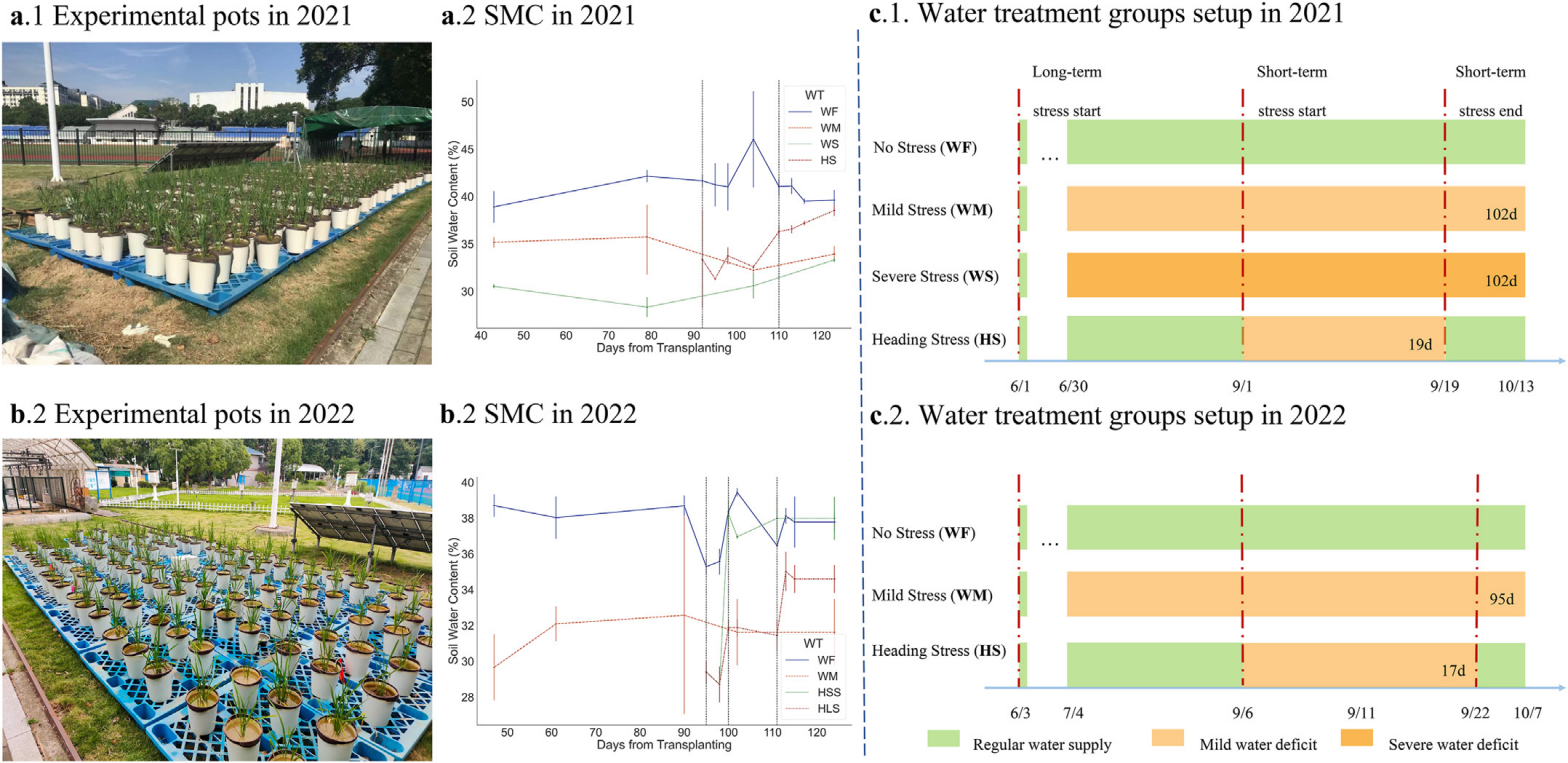
Overview of the experimental site and potted rice setup. **a**.1. Potted rice placement in 2021 with different treatment group locations randomized, **a**.2. Potted rice placement in 2022, **b**.1. Soil moisture content (SMC) monitoring in 2021, **b**.2. SMC monitoring in 2022, **c**.1. The setup of water treatments and corresponding water-deficit duration in 2021, **c**.2. The setup of water treatments and corresponding water-deficit duration in 2022. Note: Duration of water deficit (orange bars) and normal watering phases (green bars) for different treatment groups, with rewatering phases indicated by bar color change. The numbers above bars denote days of water deficit exposure.

In 2021, four water treatment groups were designed: a control group (**WF**), a group subjected to mild water stress (**WM**), a group subjected to severe water stress (**WS**) throughout the entire growth period, and a group (**HS**) exposed to a 19-day mild water stress specifically at the rice heading stage. In 2022, the **WF**, **WM**, and **HS** treatments were retained, with the HS group experiencing mild water stress over 17 days. Based on preliminary experiments, we determined the thresholds for drought severity by adjusting the soil water content to approximately 80 ​% and 60 ​% of the field moisture capacity. For the mild stress treatment, the soil water potential was maintained between −20kPa and −10kPa. The severe stress treatment involved maintaining the soil water potential in the range of −35kPa to −25kPa. The stress periods were initiated by progressively reducing the daily irrigation water until the SMC reached the target values. The rewatering time was defined as the day when the SMC recovered to the level observed in the WF group. Fig. 1a and c provide a schematic representation of the placement of potted rice and the setup of the water treatment groups, and Fig. 1b shows the SMC monitoring data of the different treatments during the conduct of the experiment. The details about the experimental setup were also provided in Supplementary Table 2.

### 2.2 Physiological traits measurement and derivation

In the experiments, multiple types of indicators were continuously monitored at different rice growth stages. Meteorological data was recorded through an independent weather station and is presented in Supplementary Fig. 1. Plant-specific indicators covered physicochemical properties and morphological structures and were extracted from 3 to 6 replicates of rice samples, serving as practical references for the imaging data analysis and model inversion outputs. For biomass-related metrics, plant samples were regularly collected, separated by organ, and weighed after drying. The leaf area (LA) was derived via a scanner (YX-1242, YAXIN). The leaf inclination angle (LIA), which is the angle between the line from the leaf base to the tip and the horizontal plane, was measured via artificial trace and computer visualization approaches. Extracts from leaf slices of specified sizes macerated in ethanol for more than 14 ​h in the dark were used to precisely quantify the concentrations of pigments, including chlorophyll *ab* (Cab) and carotenoids (Car), via a spectrophotometer (UV-2600i, Shimadzu). A handheld radiometer (YX-501, YAXIN) was used to measure the temperature (T) inside and outside the rice canopy at midday. A SPAD meter (SPAD-502PLUS, Konica Minolta) was used to measure rough chlorophyll in situ. Equivalent dry matter (*Cm*) and equivalent water content (Cw) were calculated from the fresh and dry weight and area of leaves. Table 1 lists the detailed measurement of the physiological traits. Spreading the leaf-specific traits to the canopy scale produced canopy-specific traits defined as canopy chlorophyll content (CCC), and canopy equivalent water (CEW), which are calculated by Eq. (1)**:**

CCC=Cab×LAI

(1) CEW=Cw×LAI

**Table 1:** Definitions and distributions of the PROSAIL model parameters.

Section	Parameter	Definition	Format	Distribution
**Soil**	Psoil	Soil Humidity	0-1 (wet-dry)	Uniform
	Soil_spectrum1	Preset Wet Soil	0.4–2.5 ​μm reflectance	–
	Soil_spectrum2	Preset Dry Soil	0.4–2.5 ​μm reflectance	–
**Leaf**	N	Leaf Pulp Structure	Number of heterogeneous layers	Uniform
	Cab	Chlorophyll *a b* Content		Norm
	Car	Carotenoid content		Norm
	Cw	Equivalent Water Thickness	Water content per unit area	Weibull
	*Cm*	Equivalent Dry Matter	Dry matter per unit area	Weibull
**Canopy**	LAI	Leaf Area Index		Norm
	Lidf	Leaf Angle Distribution	Campbell Parameter – Cam_alpha	Norm
	Hspot	Hot Spot Effect		Norm

Note: Psoil is not simply taken as a linear relationship based on soil moisture content. Similarly, N uses a segmented function to establish its relationship with leaf equivalent dry matter (*Cm*). A detailed description of the calculation of these parameters is shown in Supplementary Table 3.

### 2.3 Hyperspectral and front-view image acquisition and pre-processing

**Fig. 2:**
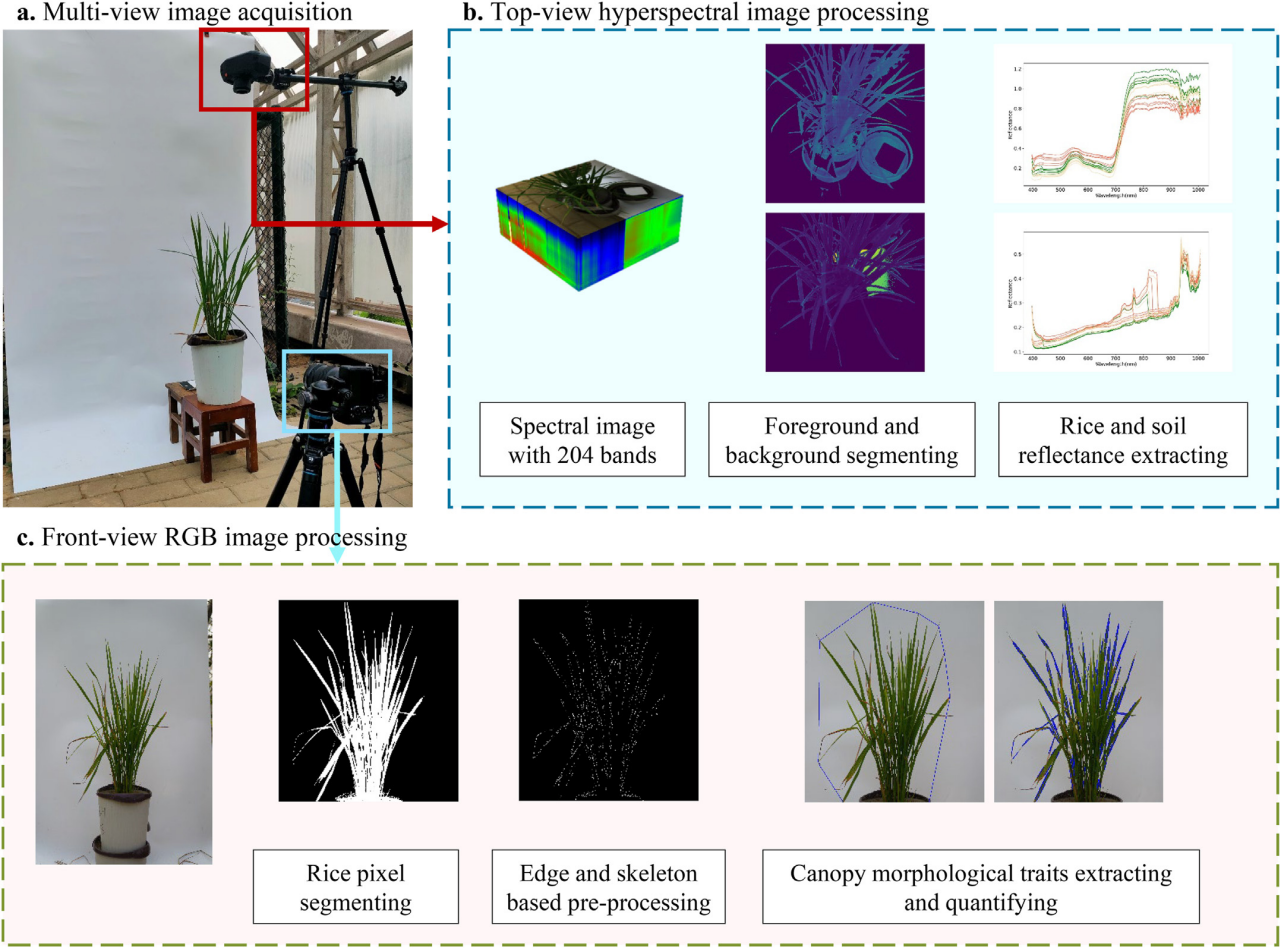
Multi-dimensional imaging data acquisition and processing flow. **a**. Image capture scene and camera layouts. **b**. Top-view hyperspectral image processing. **c**. Front-view RGB image processing.

For each sampled pot of rice, a front-view RGB image and a top-view hyperspectral image were acquired. The RGB images were captured using a SONY a7m3 digital SLR camera, operating at an exposure value of 0. The digital camera was equipped with a SEL2870 lens fixing a focal length of 28 ​mm and a field of view (FOV) of 1.53 ​× ​1.02 ​m. The hyperspectral images were procured with the SpecimIQ portable spectral camera, with a focal length of 21 ​mm and an FOV of 0.77 ​× ​0.77 ​m. The SpecimIQ provides a 3 ​nm spectral resolution and a spatial sampling resolution of 512 ​× ​512. The wavelength range spans from 397 to 1006 ​nm. Fig. 2 delineates the camera setup and image processing workflow.

a) The spectral imaging was performed perpendicularly to the ground from a fixed height of 1.8 ​m. RGB imaging was conducted horizontally, perpendicular to the plant, from a steady distance of 1.2 ​m.
b) The images were then segmented to isolate foreground and background elements. Specifically, pixels corresponding to both plant and soil were extracted. To ensure accuracy, their respective data were subsequently corrected utilizing a standard whiteboard as a reference. The mixed reflection of the plant-soil system was then calculated by averaging the reflectance values across all segmented pixels. In the process of segmentation, the Enhanced Vegetation Index (EVI) threshold was employed [37], while soil pixels were classified using the Normalized Difference Vegetation Index (NDVI) threshold [38], both of which are mathematically represented by Eq. (2):

(2) $$\text{EVI}=\frac{2.5\left(NIR-R\right)}{NIR+}$$

$$\text{NDVI}=\frac{NIR-R}{NIR+R}$$

where NIR signifies near-infrared band reflectance (895 ​nm), R signifies red band reflectance (667 ​nm), and B signifies blue band reflectance (517 ​nm).

c) The RGB images underwent processes including region-of-interest (ROI) cropping, identification and segmentation of plant subject, detection of edge and skeleton, depiction of contours, extraction of the external convex hull [[39], [40], [41], [42]]. In this step, the centroid of the plant contour was located, which determined the pixel coordinates of the plant center. The plant edges were turned into line segments by the Hough transform algorithm, and the inclination of the lines was consistent with those of the leaves. Through the above operation, the plant morphology was reduced to a geometric shape from which information related to vertical growth can be obtained. The phenotypic indicators include the relative height of the plant centroid (RHC), which represents the plant growing focus, and the distribution parameters of leaf angle, which describe vertical heterogeneity [10].

A detailed description of these indicators and parameters, along with their measurements and operational aspects, was presented in Supplementary Table 3. A biennial dataset, comprising 198 samples and over 2000 images has been assembled to support this research. The spectral python (SPy) toolkit (version 0.23.1) developed in python 3.8 was utilized in processing hyperspectral images and pixel reflectance [43].

### 2.4 Sensitivity screening and differential analysis

**Fig. 3:**
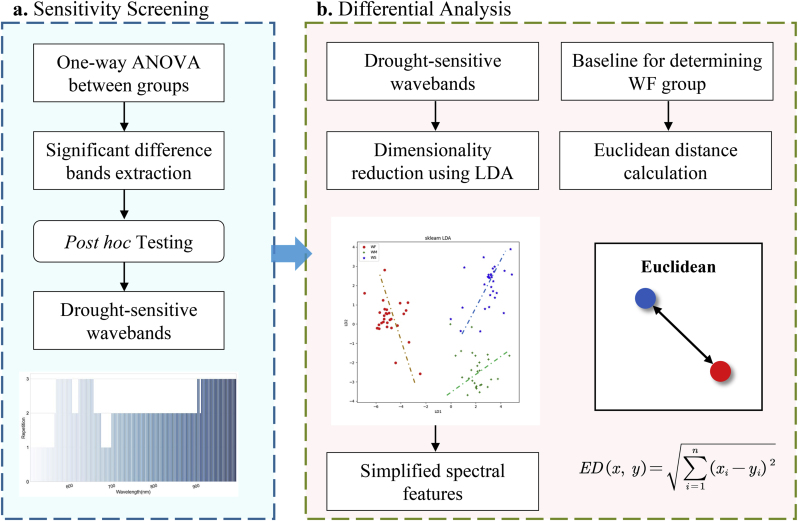
Drought-sensitive waveband screening and treatment-differential characterization. **a**. Water stress-sensitive waveband screening process. **b**. Dimensionality reduction and Euclidean distance difference analysis process.

To investigate alterations in spectral characteristics induced by varying degrees of water stress, a comprehensive analysis involving drought-sensitive waveband screening and differential assessment of rice reflectance across different treatments was conducted. The analysis process is delineated in Fig. 3. To deal with more than two treatments, a one-way ANOVA was employed to compare means of independent sample groups, aiming to assess the statistical significance of the effects exerted by varying levels of water stress on reflectance. If the ANOVA revealed the presence of at least one significant difference, Tukey's HSD (Honestly Significant Difference) post-hoc tests were subsequently conducted. These tests served to pinpoint the specific pairs of treatments between which the differences were significant while accounting for all possible group pairs and adjusting the error rate associated with multiple comparisons. Following the screening of the specific wavebands, the frequency of their significant performance was tabulated for each growth stage. The cumulative count of times each wavelength demonstrated significant differences across all sampling batches is illustrated in Fig. 3a. The sensitivity screening served as a preliminary dimensionality reduction step to highlight the effects of water stress and provide a basis for the subsequent extraction of spectral features [44].

To further refine the analysis, the linear discriminant analysis (LDA) method was employed to downscale the over 10 screened features into 2, using 70 ​% of the dataset for training purposes. The LDA method is particularly advantageous in this context, as it seeks to minimize inter-class confusion while maximizing intra-class compactness, rendering it suitable for pattern recognition tasks [45]. The within-class scatter matrix *S*_*w*_ was derived as shown in Eq. (3):

(3) $$S_{w}=\sum_{k=1}^{k}\left(\frac{\sum {x\in D_{k}}^{{xx}^{T}}}{N_{k}}-m_{k}^{2}\right)$$

where *K* is for the number of classes, *N*_*k*_ is for the number of samples, and *m*_*k*_ is for the vector of sample means after standardization. Similarly, the inter-class scatter matrix *S*_*b*_ is:

(4) $$S_{b}=\sum_{i\neq j}ij{\left(m_{i}-m_{j}\right)}^{2}$$

Subsequently, $S_{\omega }^{-1}S_{b}$ and its eigenvalues and eigenvectors are calculated, the smallest eigenvalues and the corresponding eigenvectors are selected to form a projection matrix **W**. Each feature **x**_i_ in the sample set was transformed into a new feature. $Z_{i}=W^{T}x_{i}$

The difference between groups will be evaluated by the spatial Euclidean distance. The gap of the reduced dimensional eigenvectors is obtained by Eq. (5):

(5) $$ED\left(x,y\right)=\sqrt{\sum_{i=1}^{n}{\left(x_{i}-y_{i}\right)}^{2}}$$

Considering the limitations of using ED for evaluating feature differences, it was applied to 2D (1D) features that have been downscaled by LDA, to help to identify cases of differences between water treatment groups.

### 2.5 Machine learning-based methods

**Fig. 4:**
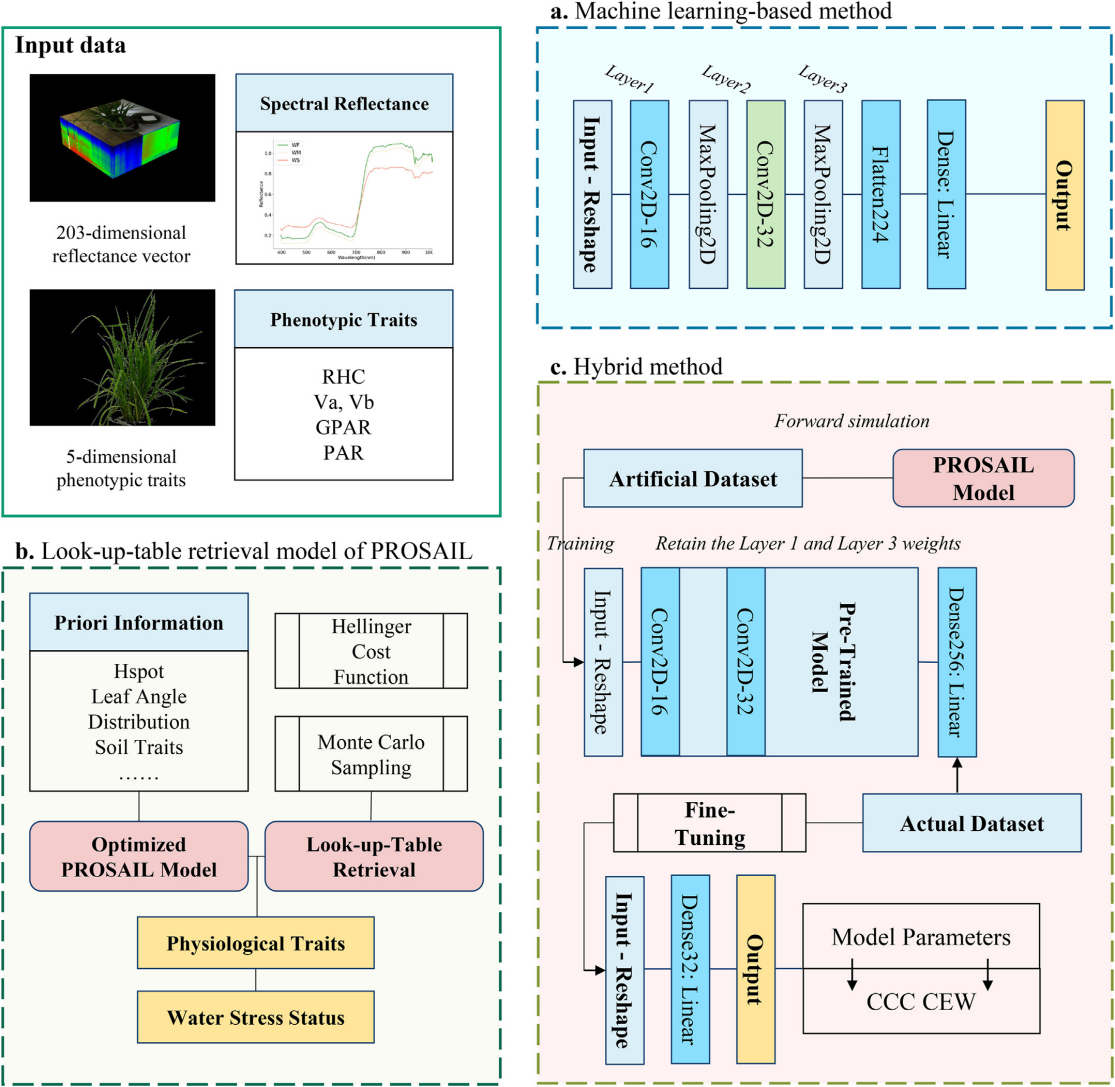
Pipeline of comparing different trait retrieval methods. **a**. Machine learning-based method using a CNN model **b**. PROSAIL model retrieval method using a look-up-table. **c**. Hybrid method.

The previously described methods for distinguishing spectral differences under water stress were mainly used for simple stress grading, but it was difficult to obtain specific traits reflecting rice water status. In this research, a machine learning regression approach was employed to learn spectral reflectance features to estimate stress specificity traits. A CNN model as shown in Fig. 4a was built to collapse the high-dimensional spectrum into the form of a two-dimensional image, extract the input data features through the convolution layers, and then predict the output through a fully connected layer. The neural network structure used in the paper was built based on TensorFlow and Keras libraries in the language Python 3.7. The PROSAIL model simulation process was run by the modified pyprosail 1.0.2 library.

### 2.6 Process-guided methods

#### 2.6.1 Retrieval model of PROSAIL

The PROSAIL parameters to be retrieved in this research are divided into soil section, leaf section, and canopy section, which are provided in Table 1. The initial reflectance corresponding to the wet and dry surfaces was defined for the properties of rice soils with high surface moisture and possibly a ponded layer. The leaf inclination angles distribution parameter – Lidf was fitted using the front-view image information and Campbell function [46]. In addition, the hot spot effect parameter – Hspot, which characterizes the ratio of leaf size to canopy height [47], was defined based on the actual experiments, and Eq. (6) was applied to define it in the model simulation:

(6) $$\text{Hspot}=\frac{leaf\;diamete}{canopy\;height}leaf\;diameter=\frac{\sqrt{leaf\;area/\left(3\times tiller\;number\right)}}{3}$$

where leaf area is measured by the leaf scanner, tiller number is the number of main tillering stems. Furthermore, the optimization of the parameters of the leaf angle distribution was based on the processing of the front-view images, and Cam_alpha was subsequently derived by fitting the Campbell function. This facilitated the pre-estimation of the range of values and the distribution pattern. Soil spectral properties were considered according to the experimental reflectance measurements of the soil surface in both flooded and dry conditions. Interpolation and fitting processes were added to form a preset reflectance of the wet and dry soil surface as the initial value for model simulation. Other parameters for PROSAIL model retrieval such as observation azimuths were used in this research with fixed values, and the way such parameters were taken and the default values were specified in Supplementary Table 4. Before proceeding with the inversion, an EFAST-based sensitivity analysis of the parameters was conducted to calculate the first-order and total-order sensitivity indices for each parameter using the primary effect values. This process aided in determining the extent to which individual parameters contribute to the simulation results, as well as assessing the impact of inter-parameter interactions on the model output.

As shown in Fig. 4b–a look-up-table (lut) was first generated using Monte-Carlo sampling based on the fitted prior distribution and parameter correlations, with each parameter combination corresponding to the reflectance data simulated by PROSAIL. Depending on the needs of the inversion, the lut contained the nine variables listed in Table 1, generating a total of 7000 groups. To establish a robust lut, the parameters were meticulously set to follow specific distributions. Among them, parameters such as LAI, Lidf, Cab, Car, and Hspot were modeled to be normally distributed, while the *Cm* and Cw parameters were configured using skewed Weibull distributions, and the Psoil and N were set to the Uniform distributions. Artificial noise was deliberately introduced to all the parameter sets, consistent with their respective distributions. Next, a cost function was established to search for the optimized parameter sets. The Hellinger function was employed in the retrieval, referring to Eq. (7):

(7) $$\text{Hel}=\sum_{i=1}^{n}{\left(\sqrt{{mea}_{i}-\sqrt{{sim}_{i}}}\right)}^{2}$$

where *mea*_*i*_ is for measured spectrum and *sim*_*i*_ is for simulated spectrum. Then, based on the trait-sensitive wavebands, the appropriate reflectance was selected for the cost value calculation. The 7000 groups of parameter sets generated by the PROSAIL model with screening wavebands were put into pre-training as artificial datasets. In the pre-training process, 70 ​% of the data was served as the training set, and 30 ​% was served as the test set. 198 groups of measured data were taken for fine-tuning, with 50 ​% as a training set and 50 ​% as a test set.

#### 2.6.2 PROSAIL-guided and image-based machine learning method

In this research, we proposed a novel hybrid method that integrates PROSAIL-guided modeling with data-driven machine learning, incorporating visual information extracted from plant images. Fig. 4a shows the multi-source input data utilized by the hybrid model, which consisted of reflectance series obtained from hyperspectral images and phenotypic traits derived through the analysis of front-view images. Initially, a pre-trained Convolutional Neural Network (CNN) model was developed using an artificial dataset generated by the optimized PROSAIL model. This dataset comprised 7000 sets of parameters and their corresponding simulated reflectance values. Subsequently, while preserving the learned weights of the convolutional layers, two additional fully connected layers were added to the CNN architecture to output new results, as depicted in Fig. 4c.

Afterward, the measured data were incorporated for fine-tuning, and five types of phenotypic indicators were combined with the reflectance data to create a synthetic dataset for predicting water stress-related traits. Pre-training in conjunction with fine-tuning is an effective training strategy for neural networks, and they are used for large-scale unlabeled data and small-scale labeled data. Since the pre-trained model has already learned the general physical associations given in the PROSAIL model, freezing the weight of some of the layers is used to preserve these features. The fine-tuning process focuses on allowing the model to learn the properties that are relevant to the particular task. The phenotypic indicators mentioned were described in detail in Supplementary Fig. 2, characterizing the vertical heterogeneity in the rice canopy. It is important to note that the input to the pre-trained model was the reflectance data generated by the modified PROSAIL model (a 203-dimensional vector of reflectance value), and the output was the physiological traits, which were derived from the PROSAIL model parameters. In the fine-tuning process, the input datasets were adjusted due to the inclusion of visual phenotypic traits in the measured data, transforming to a combination of a 203-dimensional reflectance vector and a 5-dimensional phenotypic parameter vector, and thus the input layer of the model needed to be reshaped as well as regularized. The hybrid method not only profits from the rapid convergence of the data-driven model but also incorporates multiple process functions of the radiative transfer model, which furnish the journey from perception to cognition with physical constraints. In addition, the PROSAIL inversion model and the CNN model were used as a cross-reference, with the input data being the measured spectral reflectance.

#### 2.6.3 Accuracy evaluation and error analysis

F-test was used in this research to identify the significance of differences in rice traits brought about by changing water conditions. To evaluate the accuracy of the above methods for identifying and evaluating water stress, the paper employed the correlation coefficient R, the root mean square error RMSE, the relative root mean square error RRMSE, and the general accuracy index GAI to gauge the model precision, which was calculated concerning Eq. (8):

$$R=\frac{Cov\left(mea,sim\right)}{\sigma \left(mea\right)\cdot \sigma \left(sim\right)}$$

(8) $$\text{RMSE}=\sqrt{\frac{\sum_{i=1}^{n}{\left({mea}_{i}-{sim}_{i}\right)}^{2}}{n}}$$

$$\text{RRMSE}=\frac{\text{RMSE}}{\bar{mea}}$$

GAI=1−R+RRMSE

where Cov*(mea, sim)* is for the covariance of measured and simulated values, σ(mea) is for the standard deviation, and is for the average of the measured values. GAI reflects the comprehensive performance of the model.

## 3 Results

### 3.1 Physiological response to water stress

**Fig. 5:**
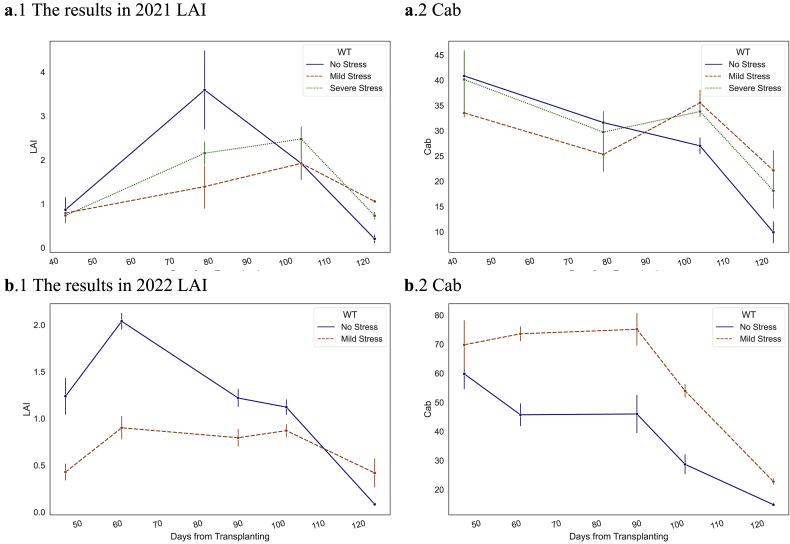
Trends and differences in LAI and Cab under long-term water stress in experiments carried out in **a**. 2021, and **b**. 2022. The x-axis represents the days from the transplant.

During the experiment, graded drought did not lead to wilting or premature senescence in the plants, but rather sustained the fundamental reproductive requirements until the harvest phase. Fig. 5 elucidates the responses of leaf area index (LAI) and Chlorophyll *a b* content (Cab) to long-term water treatments. LAI was notably influenced by long-term drought, indicating changes in rice canopy structure. During the vegetative growth period (pre-anthesis), LAI values were observed to be lower than normal. Conversely, during the reproductive growth period (post-anthesis), the disparity in LAI induced by drought diminished and even surpassed normal levels as the leaves proceeded through the senescence stage. Leaf pigmentation content quantified by Cab revealed substantial differences at the reproductive growth stage under long-term water stress, with values being higher than normal. Additionally, in the 2022 experiment, a significant difference attributable to water stress was also evident during the vegetative growth stage.

**Fig. 6:**
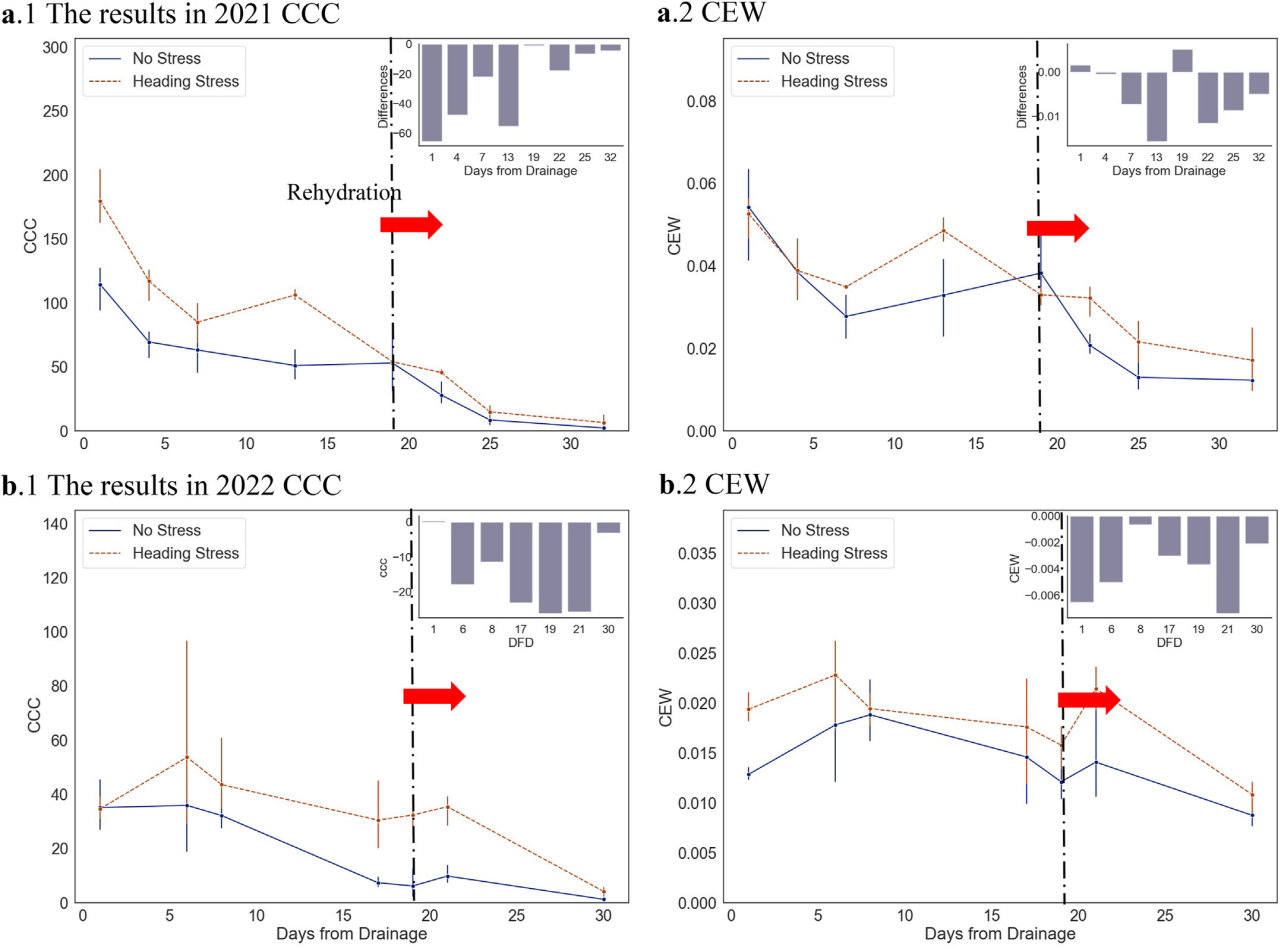
Trends and differences in CCC and CEW under short-term water stress and rehydration in experiments carried out in **a**. 2021, and **b**. 2022. The subgraphs in the figure represent the difference between the values of the WF group and the average of the stressed groups.

After anthesis in rice, tillering and the emergence cease, and to supply the development of spikes, the process of withering accelerates at this stage. As a result, both leaf area and pigment enter a phase of decline. Over the short-term drought-rehydration, neither the water content nor the leaf size exhibited significant changes, as shown in Supplementary Fig. 3. LAI, Cab, and Cw, as proxies for individual model parameters, exhibited feeble response sensitivity during the short-term drought-rehydration process. However, there was a noticeable response in the community traits of canopy leaves. Fig. 6 depicts that CCC, which indicates the chlorophyll content of the canopy, exhibited a tendency to decline as the plant underwent pigment decay. In contrast, the rate of CCC decline in plants experiencing water stress was significantly slower, resulting in a notable difference compared to controlled treatment. The gaps were observed eliminated after rehydration. Similarly, significant intergroup differences were found in CEW 7–13 days after the onset of water deficit, with these differences diminishing after rehydration. The findings suggest that mild water stress in rice after heading primarily affects leaf function rather than physiological development in the short term, and that restoration of normal flooding irrigation could facilitate functional recovery.

### 3.2 Changing trends in spectral features under water stress

**Fig. 7:**
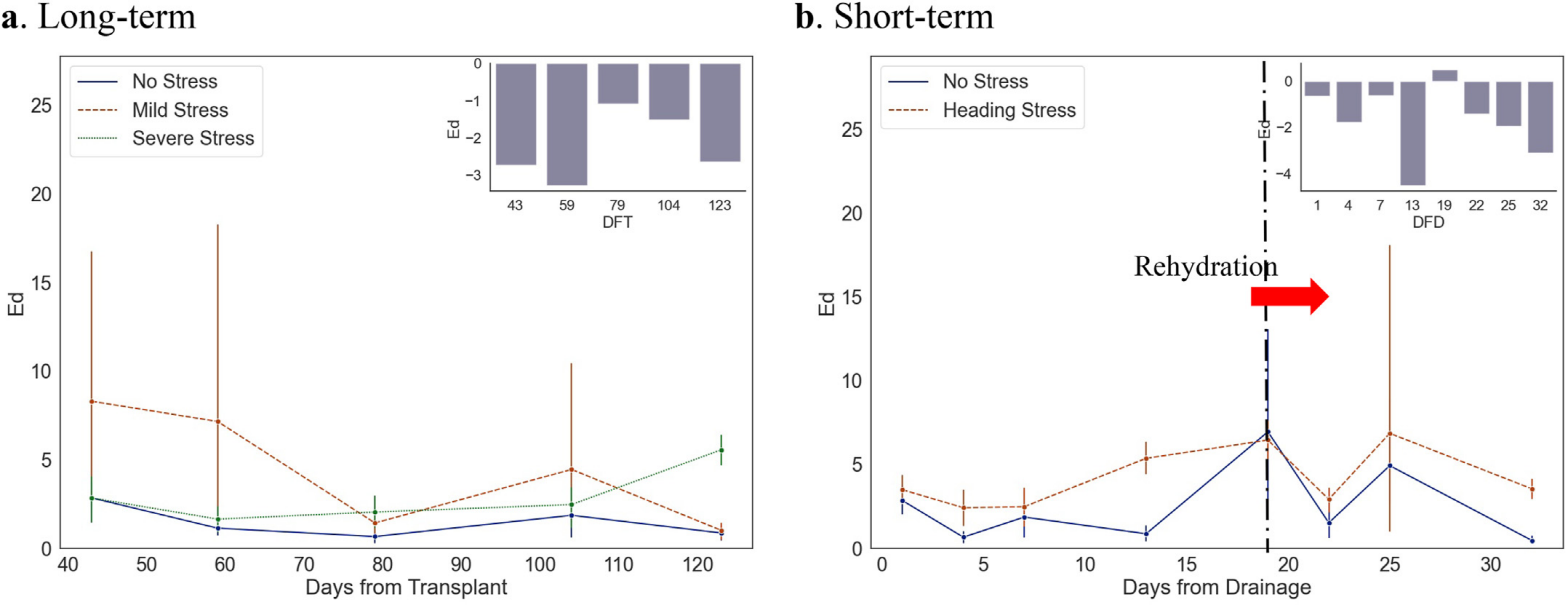
Euclidean distance gap between downscaled spectral features subjected to LDA methods under long- and short-term water stress.

The screening of sensitive wavebands revealed that the red spectral region (580–650 ​nm) and the near-infrared region (900–1000 ​nm) exhibited reflectance variations attributable to long-term water stress across multiple growth stages. Conversely, short-term drought followed by rehydration predominantly influenced the red-edge region (650–850 ​nm). Subsequently, wavebands most responsive to water variations were selected for further analysis using Linear Discriminant Analysis (LDA). To quantify differences, we employed downscaled 2D (1D) feature vectors to compute Euclidean distances (Ed) between treatment groups. Fig. 7 illustrates the disparities in Ed across various sampled growth stages. The mean eigenvectors of samples from the well-watered (**WF**) treatment group served as the baseline for stress-free conditions. Euclidean distances of other samples from this baseline were calculated using Equation (1.4) and averaged within each group. Long-term water stress primarily induced spectral differences during the nutrient growth stage. In contrast, short-term water stress elicited spectral changes within 1–4 days, which were transient and disappeared following rehydration. By computing the weighting coefficients of the individual raw wavebands in the eigenvectors obtained by LDA, the significance of different wavelength ranges can be evaluated. After thorough analysis and screening, it has been determined that the spectral reflectance within the range of 742–841 ​nm exhibited optimal indicative properties for assessing drought impacts. Consequently, this specific spectral range was utilized for the inversion process, whereas dissolved information was excluded. The statistical analysis of spectral reflectance was conducted to generate precise and pertinent input reflectance data for the retrieval method, while simultaneously validating the efficacy of spectral features in differentiating various types of water stress.

### 3.3 Improvement in the derivation of canopy-specific traits

**Fig. 8:**
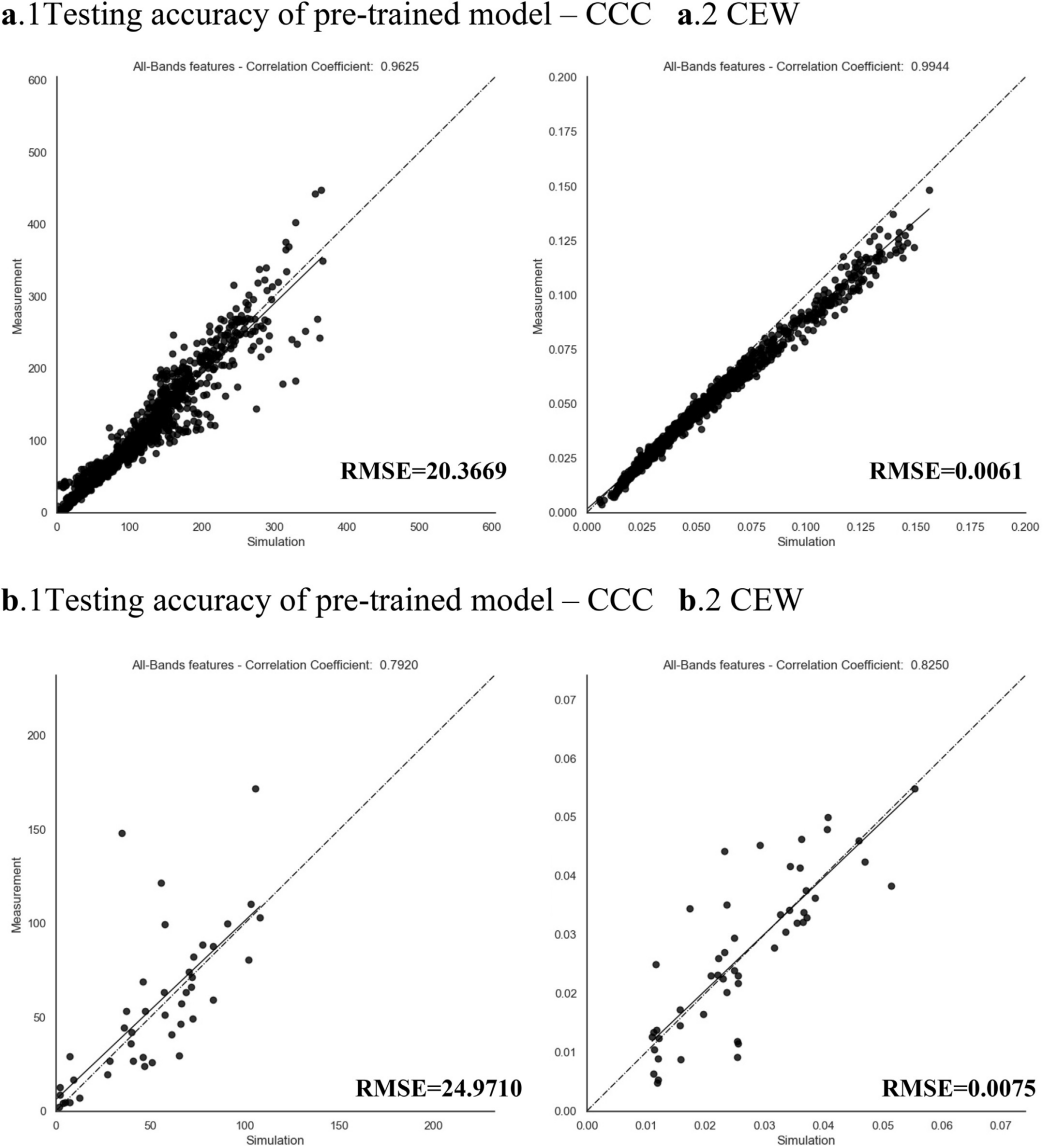
Test accuracy of canopy-specific traits using hybrid method retrieval. **a**. Testing results of the pre-trained model. **b**. Testing results of the fine-tuned model.

The experimental results suggest that derived canopy-specific traits (CCC and CEW) based on LAI and leaf-specific traits were more effective in indicating the impacts of short-term drought-rehydration events. The ability of the hybrid method to invert derived traits was tested in this study. Fig. 8 presents the test accuracies for CCC and CEW during pre-training and fine-tuning processes. Based on the virtual dataset generated by the PROSAIL model, the results demonstrate high accuracy for CCC (R ​= ​0.9625, RMSE ​= ​20.3669 ​μg ​cm^−2^) and CEW (R ​= ​0.9944, RMSE ​= ​0.0061 ​cm). Subsequently, the fine-tuning process based on the measured dataset was validated, also achieving good accuracy for CCC (R ​= ​0.7920, RMSE ​= ​24.971 ​μg ​cm^−2^) and CEW (R ​= ​0.8250, RMSE ​= ​0.0075 ​cm).

**Fig. 9:**
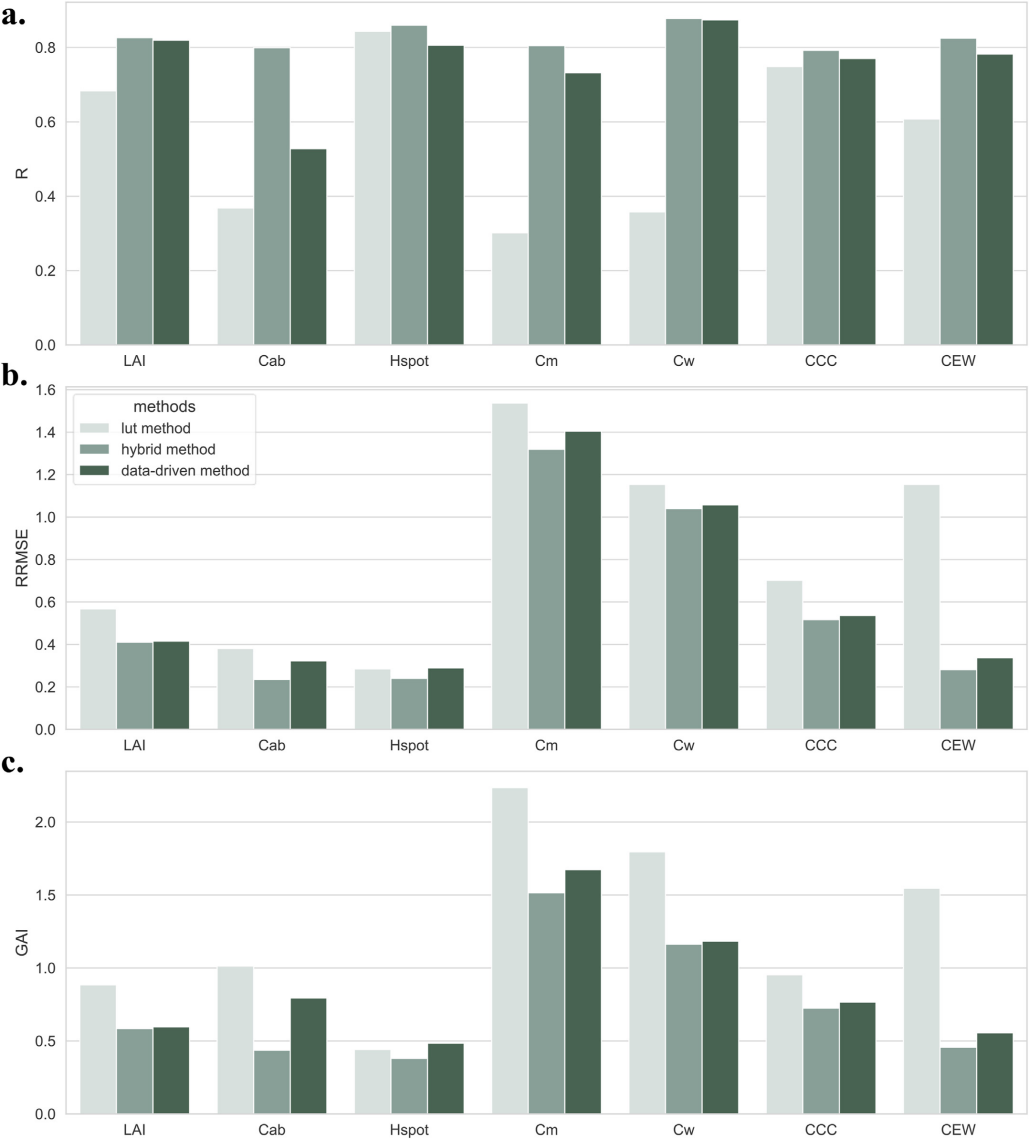
Comparison of the accuracy of hybrid and other methods for retrieving rice physiological and morphological traits. **a**. The results of R. **b**. The results of RRMSE. **c**. The results of GAI.

To assess the improvement of the hybrid method in retrieving physiological traits and evaluating water stress, its performance was juxtaposed against two other prevalent methods. The comparison is based on the evaluation index of R, RRMSE, and GAI is depicted in Fig. 9. For individual traits, including LAI, Cab, Hspot, *Cm*, and Cw - which also serve as parameters in the PROSAIL model - the hybrid method exhibited the best accuracy, followed by the data-driven method. Regarding the two canopy-specific traits, the hybrid method demonstrated similarly impressive performance. Specifically, the RRMSE for calculating CCC was 0.5168, marking a reduction of 18.51 ​% compared to the lut method and a decrease of 3.65 ​% compared to the data-driven method. The RRMSE for calculating CEW is 0.2811, which was a decrease of 75.63 ​% compared to the lut method and a decrease of 16.66 ​% compared to the data-driven method. The detailed values of the evaluation index are listed in Supplementary Table 6. In the results, the lut method performed poorly in the estimation of leaf-scale traits such as Cab, Cw, and *Cm*. Consequently, this led to difficulties in accurately retrieving CCC and CEW. The deficiency could be attributed to the properties of the PROSAIL model, which merely considers Cw and *Cm* as measures of whether or not the overall plant water content and dry matter are impaired. However, studies have demonstrated that low-intensity drought does not induce significant physiological alterations at the leaf scale, instead, it primarily influences canopy structure and water vapor flux. The substantial discrepancy between the scale of the model application and the experimental design scale emerged as the main factor contributing to the lut method's lack of precision. Some leaf-specific traits are inherently challenging to invert accurately. In contrast, alternative models based on machine learning have proven effective in reducing these errors.

## 4 Discussion

### 4.1 Water stress impact evaluation combined with physiological traits and spectral signatures

The impact of drought on crops is determined by its occurrence period, severity, and duration. Water stress exerts varying effects at different rice growth stages from emergence to maturity [48]. Before anthesis, water deficit inhibits tillering and leaf expansion, whereas after anthesis, it inhibits spike ripening [49]. Prolonged and severe drought can cause rapid dehydration and wilting in rice plants, whereas mild drought elicits a transient resistance response in plants [50]. Once rice enters the reproductive growth stage, it becomes highly susceptible to drought, which affects yield formation [51]. By comparing rice traits under long- and short-term drought, a shift from antagonistic to avoidance strategies can be observed [52] Short-term water deficit stimulates drought tolerance in rice by boosting photosynthetic efficiency and minimizing the loss of assimilates through adaptive mechanisms, such as altering leaf inclination to a more erect position. So it was possible to witness the maintenance of CCC and CEW. In contrast, entering a prolonged water deficit situation, rice growth was retarded and the leaf area contracted, providing only the assimilated products necessary for survival. Changes in water conditions within paddies are complex and challenging to estimate, where both immediate and delayed effects of water stress must be considered for accurate assessment. To address this, our research encompassed experiments with both long- and short-term drought treatments, designed to mimic water conditions typically encountered in real-world water-saving irrigation practices. The findings reveal significant variations in the impacts of water stress across different growth stages of rice.

This necessitated a targeted approach to identifying and evaluating water status during specific periods. However, sampling and analyzing plants every time they enter a new growth stage would increase the workload. Through experimentation, we verified that canopy traits, exemplified by CCC and CEW, effectively indicate the impacts of short-term water stress. While traits such as LAI and leaf angle distribution, which are components of the PROSAIL model inputs, can be directly inverted, they exhibit limited sensitivity to water stress. SPAD is a widely used optical indicator for assessing leaf chlorophyll status. During short-term drought-rehydration process, SPAD was observed higher than usual, a trend that aligned with CCC (Supplementary Fig. 4). However, the measurement of SPAD necessitates specialized instruments, making it less convenient compared to the CCC indicator. Another commonly employed indicator for canopy water status is the Temperature Difference between the outside and inside canopy (TD). Under short-term drought, TD was observed lower than normal conditions. When watering was resumed, the temperature gap was eliminated. Despite these, the results obtained for TD did not exhibit statistically significant credibility, thus precluding its use as a standalone indicator for the onset and termination of water stress. CCC and CEW emerged as valuable markers for short-term drought-rehydration states, as they can be directly derived from model parameters. Their sensitivity to water changes and detailed portrayal of rice physiology make these canopy traits highly valuable for application in water stress identification and evaluation. Furthermore, CCC and CEW integrate leaf area, pigmentation, and organ water content, offering a holistic perspective on the rice's physiological status.

### 4.2 Improvement of model simulation based on image-based leaf angle distribution parameters

**Fig. 10:**
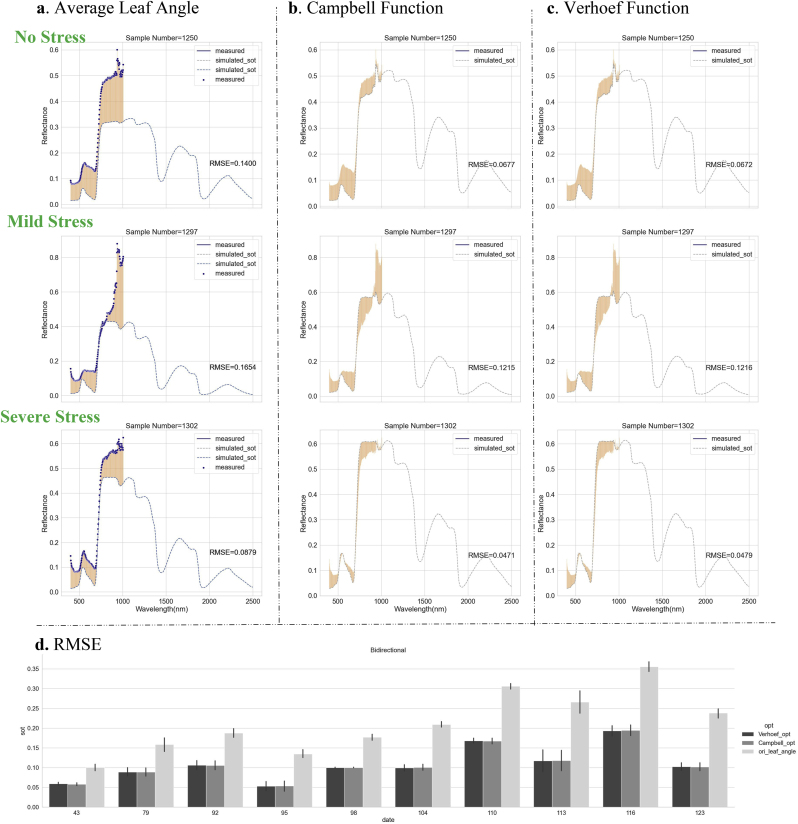
Comparison of simulated results with measured reflectance when different forms of leaf angle parameters were used as model inputs. **a**. Using average leaf angle. **b**. Using the fitted Campbell distribution function parameter – Cam_alpha. **c**. Using the fitted Verhoef 2-parameter distribution function parameters – Va, Vb. **d**. RMSE value calculated with measured reflectance at the corresponding wavelengths. In subgraphs **a**, **b**, and **c**, the blue scatter marks the measured reflectance values and the dotted line marks the simulated reflectance values with the orange bar marking the difference between the two.

Crop morphological phenotypes are pivotal for analyzing physiological traits, growth status, and their specific responses to water stress. In this study, we provided a comprehensive description of rice phenotypes, taking into account various distribution patterns of the leaf inclination angle (denoted as Cam_alpha), the manifestation of the hotspot effect (Hspot), and the localization of the growth focus (RHC). Within the PROSAIL model, the leaf angle distribution (LAD) is recognized as a significant influencing factor. The outcomes of the first-order and total-order global sensitivity analyses for certain model parameters are presented in Supplementary Fig. 5. These results underscore the crucial role of LAD in the reflectance simulation process. The acquisition and processing of front-view images facilitated the high-precision extraction of leaf angles, eliminating the need for laborious manual measurements. Consequently, the LAD parameters within the PROSAIL model were based on measured values tailored to the unique characteristics of each plant, rather than relying on empirical or typical values. Furthermore, a mere averaging of angles fails to adequately capture the leaf morphology within the canopy. In contrast, the parameters of fitted distribution functions can aid in simulating radiative transfer processes. As schematized in Fig. 10, better reflectance simulation results were obtained using image-based leaf angle distribution parameters over multiple observations throughout the growth cycle. The comparison is between the reflectance values simulated by the PROSAIL model and those measured by the SpecimIQ camera. The RMSE averaged 0.0985, representing a significant reduction of 41.12 ​% compared to the original method that rely on the fixed mean leaf angle (RMSE ​= ​0.1673). Upon comparing the two distribution forms, their performance was comparable. Therefore, we opted for the Campbell function, which has fewer parameters, and defined its distribution parameter as Cam_alpha for the forward simulation of the PROSAIL model.

### 4.3 Synergistic effects of multi-view imaging in vegetation spectral analysis

The effectiveness of the hybrid method can be attributed to the integration of data-driven and radiative transfer model (RTM) approaches, facilitating rapid iteration and the establishment of physical connections. More fundamentally, phenotypic indicators that are highly indicative of rice canopy-specific traits were extracted and utilized in the fine-tuning of the model. This was achieved through the effective exploitation and utilization of visual phenotypic information. The application of computer vision techniques has enabled the acquisition of high-throughput and cost-effective data for plant monitoring. Numerous studies have explored the use of image features to reflect plant physiological states [53]; [39,54]. The current methods in computer vision utilized for plant phenotyping still describe plant morphology in an abstract and geometric way. Despite the capability of 3D reconstruction and point cloud acquisition to portray intricate plant details, their associated costs remain high. Although not as informative as the multi-view imaging, this study provided a simple method for extracting plant traits from both top and front views, to explore their potential for synergy with spectral features. Multidimensional imaging data can provide complementary information in the assessment of complex water stress traits. The information contained within images is extensive, yet there is currently no standardized parameterization method. One of the challenges in current research is to eliminate redundant noise and extract interpretable features.

## 5 Conclusion

Multidimensional remote sensing has been widely applied in agricultural monitoring and drought evaluation, yet its accuracy and simplicity fall short of precision agriculture water management needs. The advent of distributed phenotyping platforms and imaging devices has generated vast amounts of crop data. This research aims to use ground-based observations to evaluate water stress in rice, capturing comprehensive crop responses. The proposed CCC and CEW traits revealed short-term drought-rehydration impacts during reproductive growth period. Multidimensional imaging data was used to establish relationships between image properties and rice phenotypes, exemplified by RHC, which characterizes aboveground growth focus. Phenotypic indicators and spectral signatures were integrated for a comprehensive description of water stress impacts. The integration of PROSAIL and CNN models enhanced water stress evaluation. Future work will develop an extensive rice stress-related trait library, using standardized image processing and hybrid methods that combine physical knowledge with data-driven models for accurate water stress identification and quantification.

## Data availability

The data that support this study are available on request from the corresponding author.

## Declaration of competing interest

The authors declare that they have no known competing financial interests or personal relationships that could have appeared to influence the work reported in this paper.
