# Supplementary material for: A hybrid method for water stress evaluation of rice with the radiative transfer model and multidimensional imaging
Source: Plant Phenomics. 2025 Feb 28;7(1):100016. doi: 10.1016/j.plaphe.2025.100016 (PMC12709993; doi:10.1016/j.plaphe.2025.100016)
Supplement: Multimedia component 1 [file mmc1.docx]

Supplementary Tables

**Supplementary Table 1.** The details in the experiment setup

| **Experimental group** | **Water stress level** | **Duration of flood irrigation** | **Duration of water stress** | **Sampling date** |
| --- | --- | --- | --- | --- |
| **Experiment in 2021** | | | | |
| **WF** | No stress | Day 1 to 134 | \ | Day 43, 59, 79, 92, 95, 98, 104, 110, 113, 116, 123 |
| **WM** | Mild stress | Day 1 to 25 | Day 25 to 134 | Day 43, 59, 79, 104, 123 |
| **WS** | Severe stress | Day 1 to 25 | Day 25 to 134 | Day 43, 59, 79, 104, 123 |
| **HS** | Mild stress at Heading Stage | Day 1 to 92  & Day 111 to 134 | Day 92 to 110 | Day 92, 95, 98, 104, 110, 113, 116, 123 |
| **Experiment in 2022** | | | | |
| **WF** | No Stress | Day 1 to 126 | \ | Day 47, 61, 90, 95, 98, 100, 102, 111, 113, 115, 124 |
| **WM** | Mild Stress | Day 1 to 20 | Day 20 to 126 | Day 47, 61, 90, 102, 124 |
| **HS** | Mild Stress at Heading Stage | Day 1 to 90 & Day 107 to 126 | Day 90 to 107 | Day 95, 100, 102, 111, 113, 115, 124 |

Note: The number of days in Table 1 refers to the days from rice transplanting. In 2021, Day is June 1^st^. In 2022, Day 1 is June 3^rd^.

**Supplementary Table 2.** Measurement of soil physical and chemical properties

| **Clay proportion %** | **Silt proportion %** | **Sand proportion %** | **Soil bulk density (g⋅cm^-3^)** | **Soil electric conductivity(us⋅cm^-1^)** | **Saturated water content %** | **Field Capacity %** | **Absorption moisture content %** |
| --- | --- | --- | --- | --- | --- | --- | --- |
| 15.15 | 70.75 | 14.09 | 1.39 | 82.11 | 32.12 | 30.52 | 5.07 |

Note: The water stress levels in this study were developed based on soil mositure characteristics measured beforehand, under which we ensure the survival and growth of the rice.

**Supplementary Table 3.** Several rice physiological and morphological traits and their measurements in the experiment.

| **Types** | **Traits** | **Nomenclature** | **Measurements** | **Units** |
| --- | --- | --- | --- | --- |
| Manual Measurement | Plant Height | PH | Tapeline | cm |
|  | Tillering amount | F | Manual counting | \ |
|  | Leaf Area Index | LAI | Leaf area scanner | m^2^ ⋅m^-2^ |
|  | Leaf Angle Distribution | ALA | Computer vision methods and distribution function fitting | degree |
|  |  | Cam_alpha |  | \ |
|  |  | Va |  | \ |
|  |  | Vb |  | \ |
|  | Leaf Pigment | Cab | Spectro-photometer | μg⋅cm^-2^ |
|  |  | Car |  | μg⋅cm^-2^ |
|  |  | Cm | Dry and weight | g⋅cm^-2^ |
|  |  | Cw |  | cm |
|  | Hotspot Effect Parameter | Hspot | Eq. 2.1 | \ |
|  | Leaf Structure Parameter | N | Derived by *Cm* | \ |
|  | Soil Moisture Content | SMC | Dry and weight | \ |
|  | Greenness Ratio | GPAR | Refer to Supplementary Figure 1 | \ |
|  | Relative Height of Centroid | RHC |  | \ |
|  | Relative Contour Size | PAR |  | \ |

Note: The leaf structure parameter N is derived by Cm and the follow Eq and graph:


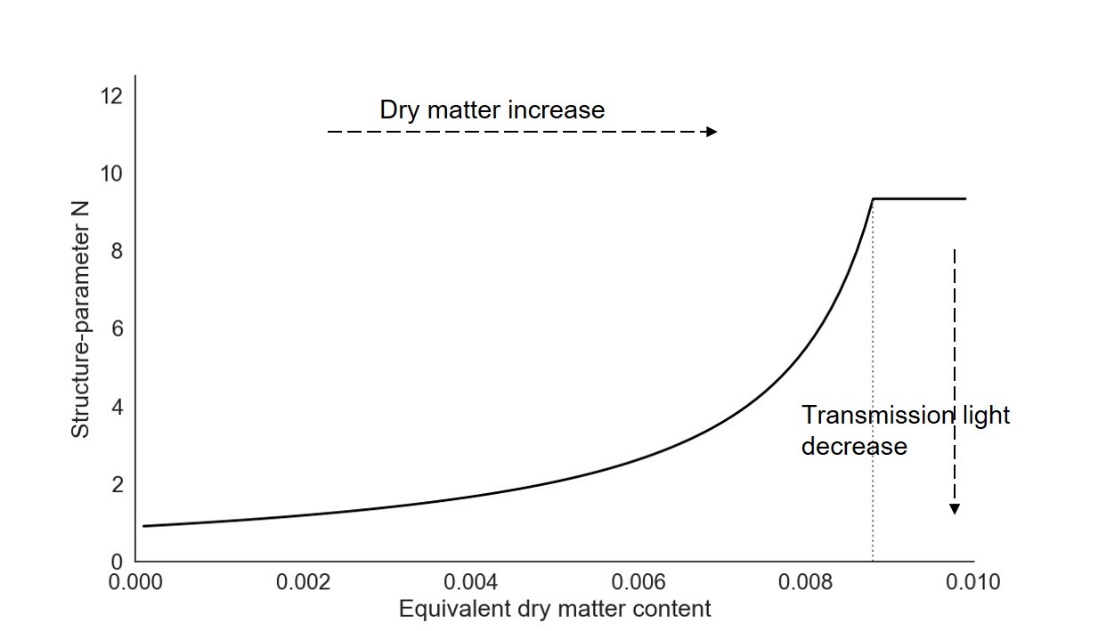


The soil humidity parameter Psoil is derived by SMC and the follow Eq and graph:


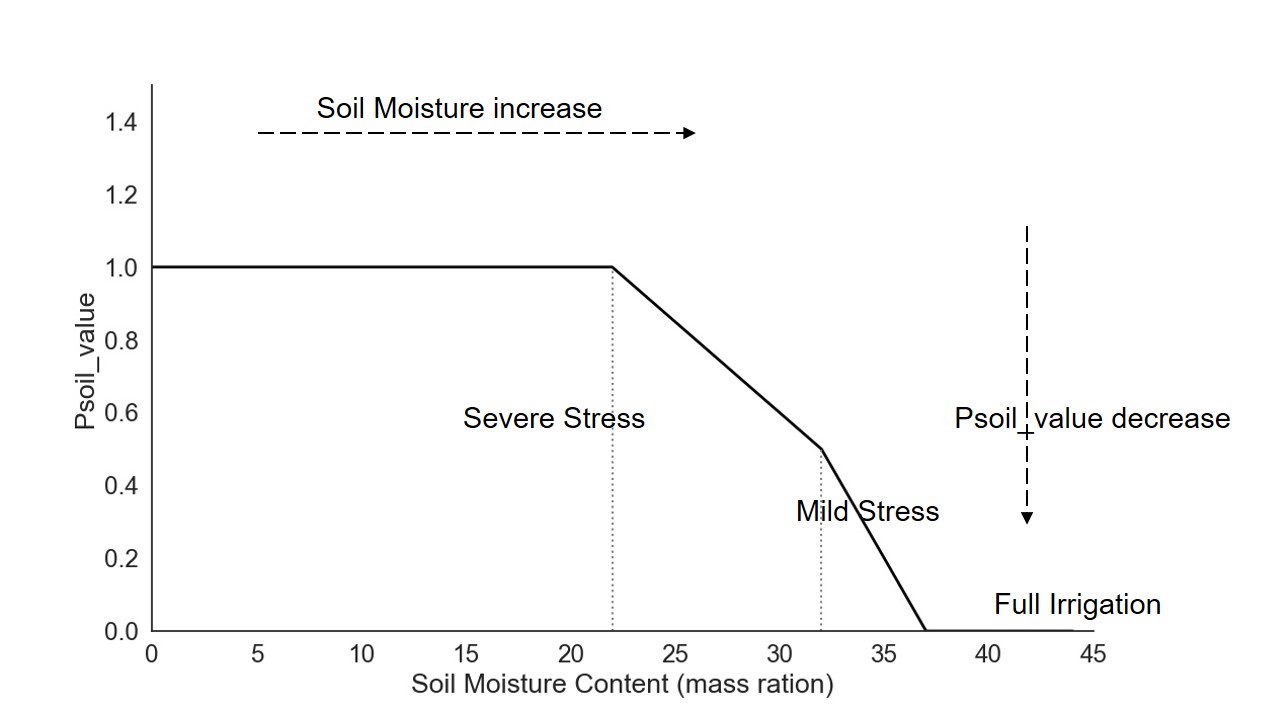


**Supplementary Table 4.** Default value settings for a section of parameters in the PROSAIL model.

| **Parameters** | **Nomenclature** | **Default value** | **Units** |
| --- | --- | --- | --- |
| Type of lead angle distribution | typelidf | 2 | 1 or 2 |
| Solar zenith angle | tts | 60 | degree |
| Sensor zenith angle | tto | 30 | degree |
| Relative sensor-solar azimuth angle | psi | 330 | degree |
| Anthocyanins content | ant | 0 | g⋅cm^-2^ |
| The brown/senescent pigment | cbrown | 0 | - |
| The alpha angle used in the surface scattering calculation | alpha | 40 | degree |
| Soil scalar 1 (brightness) | rsoil | 1 | - |
| The type of output reflectance | factor | SDR | - |

Note: Parameters related to the solar angle were calculated by reading the exif information of the images, while the sensor angle was fixed by the tripod. The exif information of the images includes the moment of capture (year-month-day-hour-minute-second), the location (latitude and longitude), and the solar altitude and azimuth at that moment could be subsequently derived using Pysolar library (version 3) based on python 3.7.

**Supplementary Table 5.** Testing set precision of the hybrid method in pre-training and fine-tuning procedures

| **Evaluation Index** | **CCC** | **CEW** |
| --- | --- | --- |
| Pre-trained R | 0.9625 | 0.9944 |
| Pre-trained RMSE | 20.3669 | 0.0061 |
| Fine-tuned R | 0.7920 | 0.8250 |
| Fine-tuned RMSE | 24.9710 | 0.0075 |
| Fine-tuned RRMSE | 0.5168 | 0.2811 |
| Fine-tuned GAI | 0.7248 | 0.4561 |

**Supplementary Table 6.** Statistics on the precision of different methods for estimating water stress traits

| **Evaluation Index** | **Type of**  **Methods** | **Canopy Traits** | | **Individual Traits** | | | | | |  |
| --- | --- | --- | --- | --- | --- | --- | --- | --- | --- | --- |
|  |  | CCC | CEW | | LAI | Cab | Hspot | Cm | Cw | |
| R | lut | 0.7487 | 0.608 | | 0.6834 | 0.3685 | 0.8432 | 0.3013 | 0.3579 | |
|  | hybrid | **0.792** | **0.825** | | **0.8263** | **0.7993** | **0.8599** | **0.8047** | **0.8778** | |
|  | data-driven | 0.7703 | 0.7822 | | 0.8192 | 0.5277 | 0.806 | 0.7319 | 0.874 | |
| RMSE | lut | 72.0861 | 0.0326 | | 1.8693 | 10.5155 | 0.004 | **0.0245** | **0.0315** | |
|  | hybrid | **24.971** | **0.0075** | | **0.6323** | **6.3602** | **0.0032** | 0.0269 | **0.0348** | |
|  | data-driven | 25.9191 | 0.0091 | | 0.6401 | 8.7287 | 0.0039 | 0.0286 | 0.0354 | |
| RRMSE | lut | 0.7019 | 1.1537 | | 0.5675 | 0.381 | 0.2851 | 1.5368 | 1.1537 | |
|  | hybrid | **0.5168** | **0.2811** | | **0.4103** | **0.2347** | **0.24** | **1.3191** | **1.0398** | |
|  | data-driven | 0.5364 | 0.3373 | | 0.4154 | 0.3221 | 0.2893 | 1.4049 | 1.057 | |
| GAI | lut | 0.9532 | 1.5457 | | 0.8841 | 1.0125 | 0.4419 | 2.2355 | 1.7958 | |
|  | hybrid | **0.7248** | **0.4561** | | **0.584** | **0.4354** | **0.3801** | **1.5144** | **1.162** | |
|  | data-driven | 0.7661 | 0.5551 | | 0.5962 | 0.7944 | 0.4833 | 1.673 | 1.183 | |
