# Supplementary material for: A hybrid method for water stress evaluation of rice with the radiative transfer model and multidimensional imaging
Source: Plant Phenomics. 2025 Feb 28;7(1):100016. doi: 10.1016/j.plaphe.2025.100016 (PMC12709993; doi:10.1016/j.plaphe.2025.100016)

a. The record of Radiance during the two-year experiment

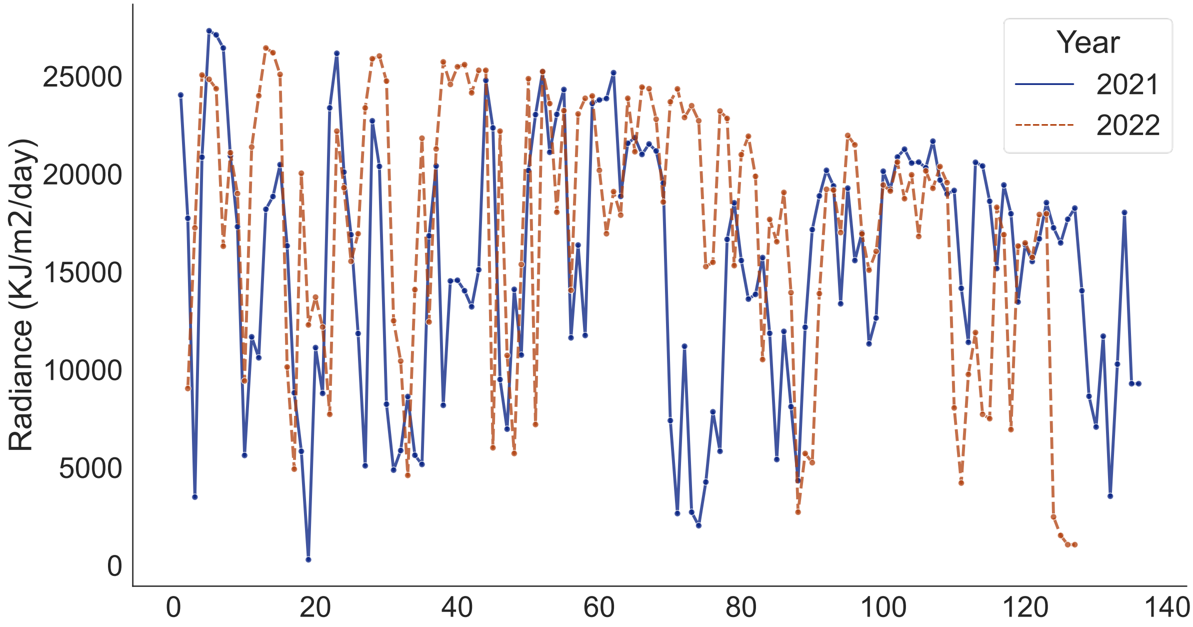

b. The record of Daily Temperature during the two-year experiment

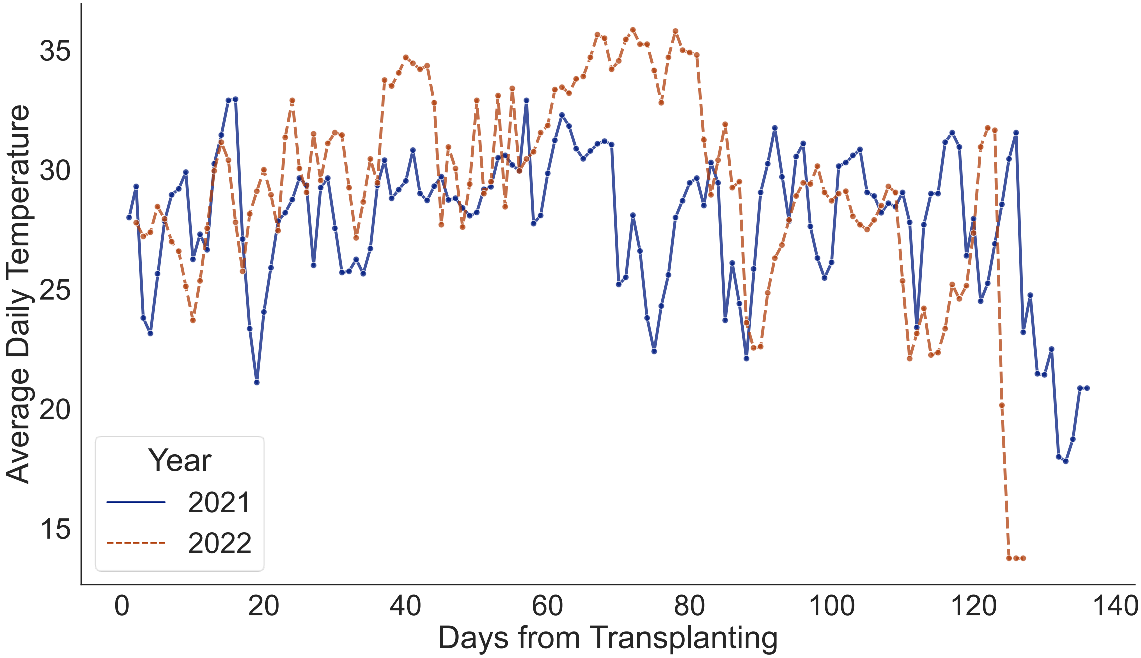

a. Traits extracted from the outline of the plant

GPAR

$$\frac{\text{Greenness Projected Plant Area}}{\text{Total Projected Plant Area}}$$

PAR

$$\frac{\text{Plant Perimeter}}{\text{Total Projected Plant Area}}$$

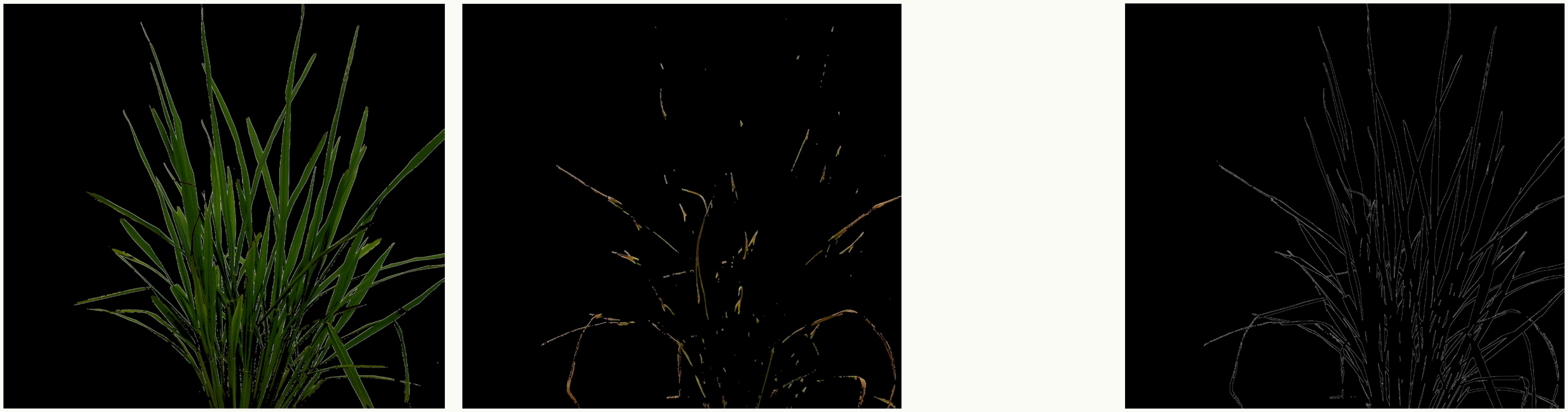

b. Traits extracted from the vacuated skeleton of the plant

RHC

$$\frac{\text{Centroid Height (pixels)}}{\text{Plant Height (pixels)}}$$

Verhoef leaf angle distribution parameters

$$V(\theta_l) = \frac{2\left(a \cdot \sin 2\theta_l + \frac{1}{2} b \cdot \sin 4\theta_l + \theta_l\right)}{\pi}$$

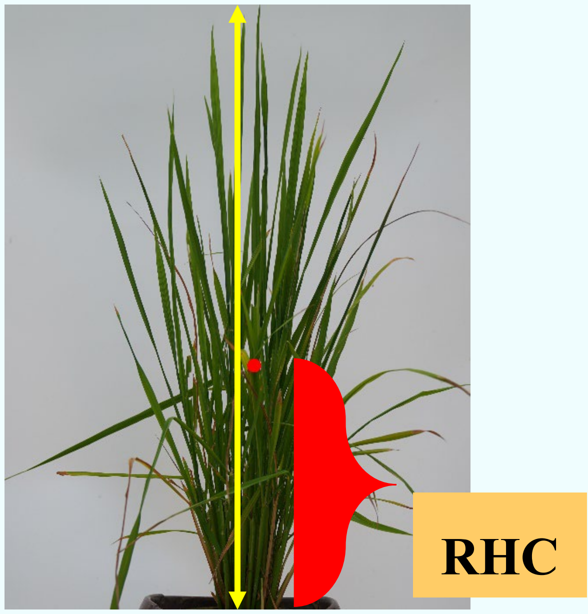

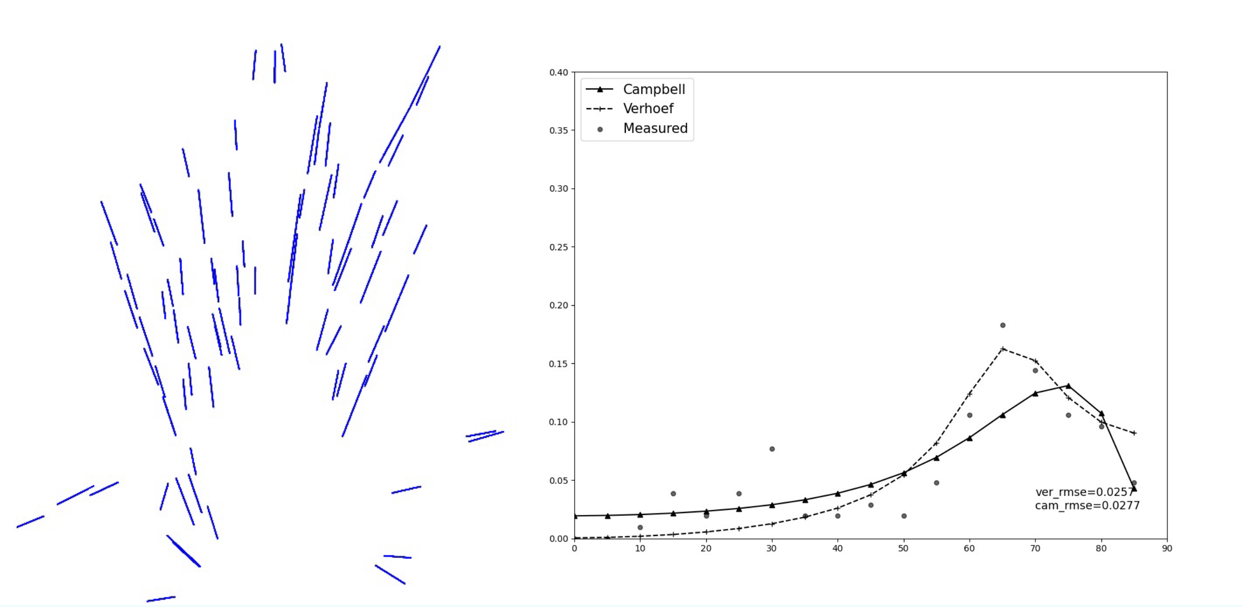

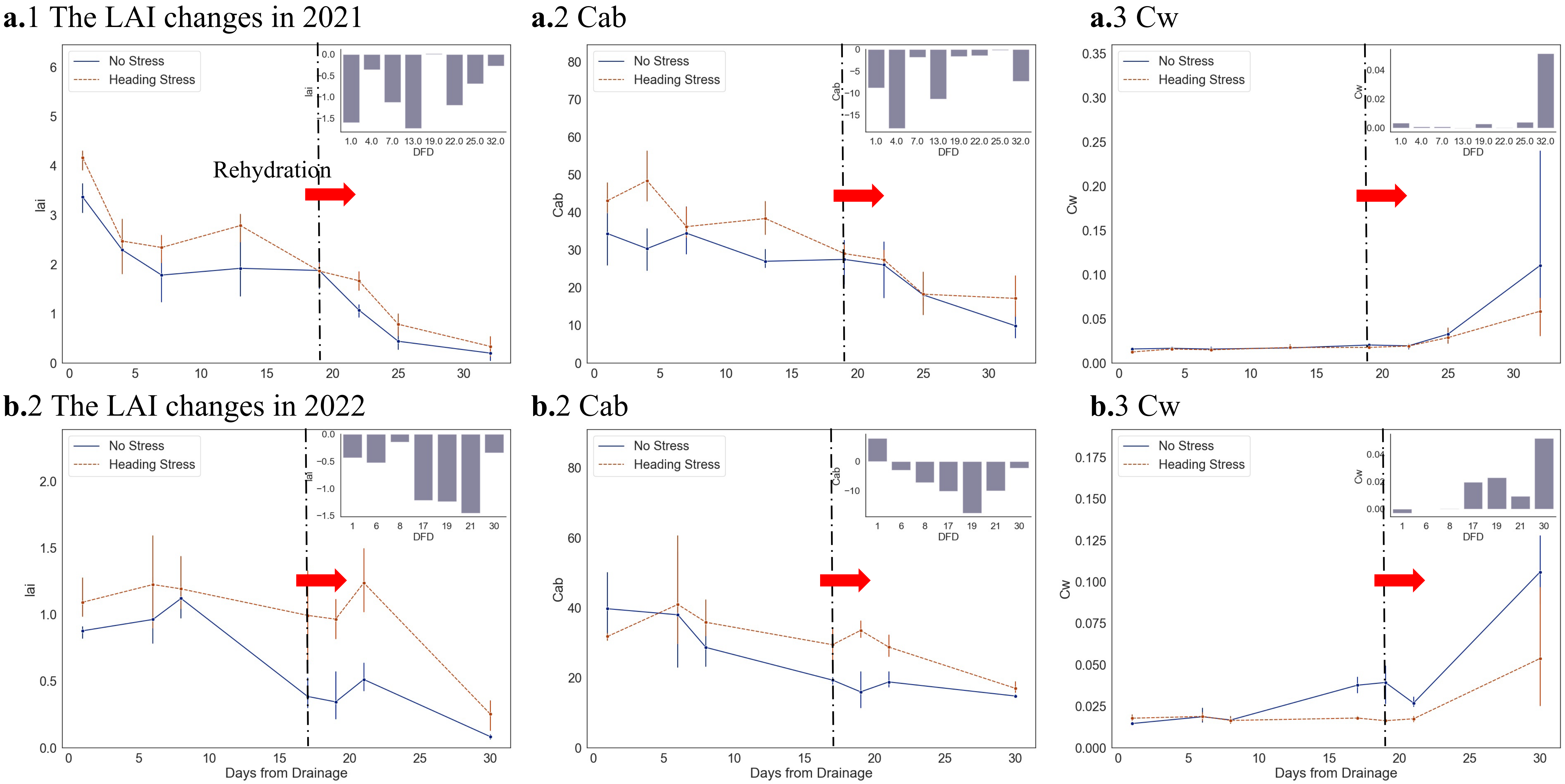
a.1 The LAI changes in 2021
a.2 Cab
a.3 Cw
b.2 The LAI changes in 2022
b.2 Cab
b.3 Cw
No Stress
Heading Stress
Rehydration
lai
Cab
Cw
DFD
Days from Drainage

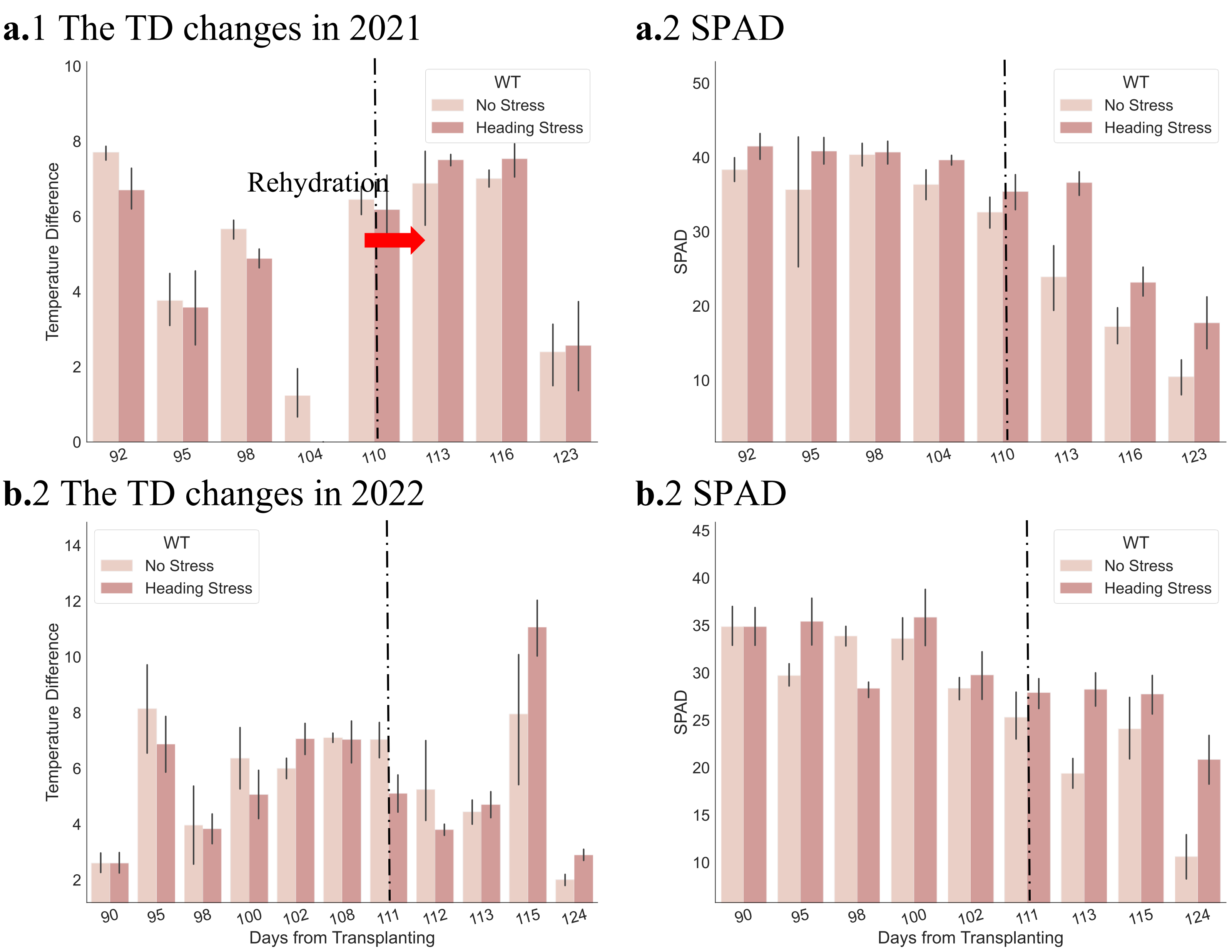
a.1 The TD changes in 2021
Rehydration
Temperature Difference
WT
No Stress
Heading Stress
a.2 SPAD
SPAD
b.2 The TD changes in 2022
Temperature Difference
Days from Transplanting
b.2 SPAD
SPAD
Days from Transplanting

a. First-order sensitivity analysis of PROSAIL model parameters

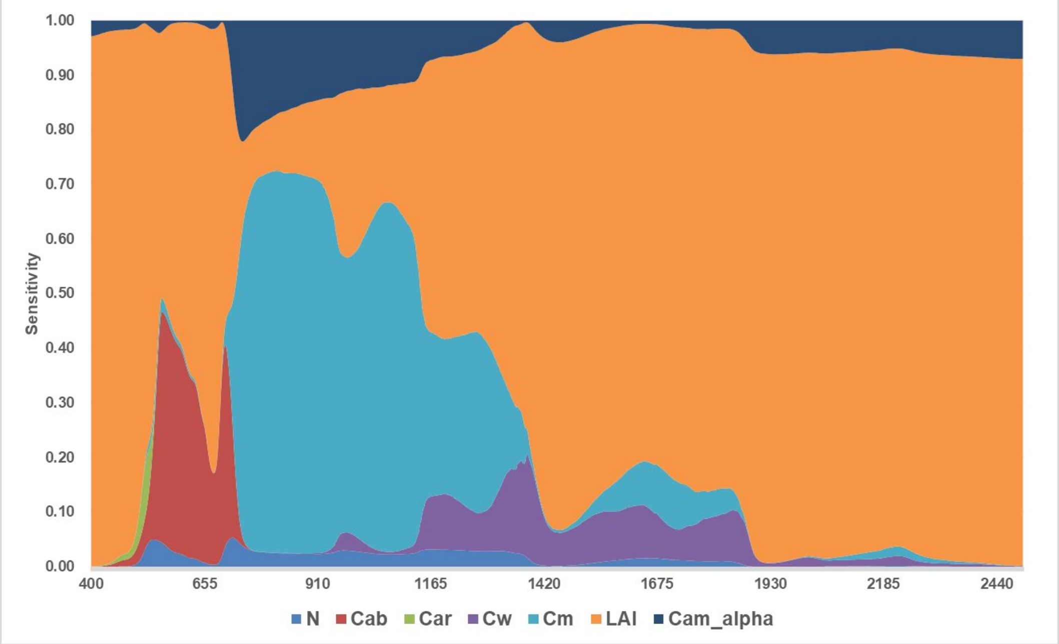

b. Total-order sensitivity analysis of PROSAIL model parameters

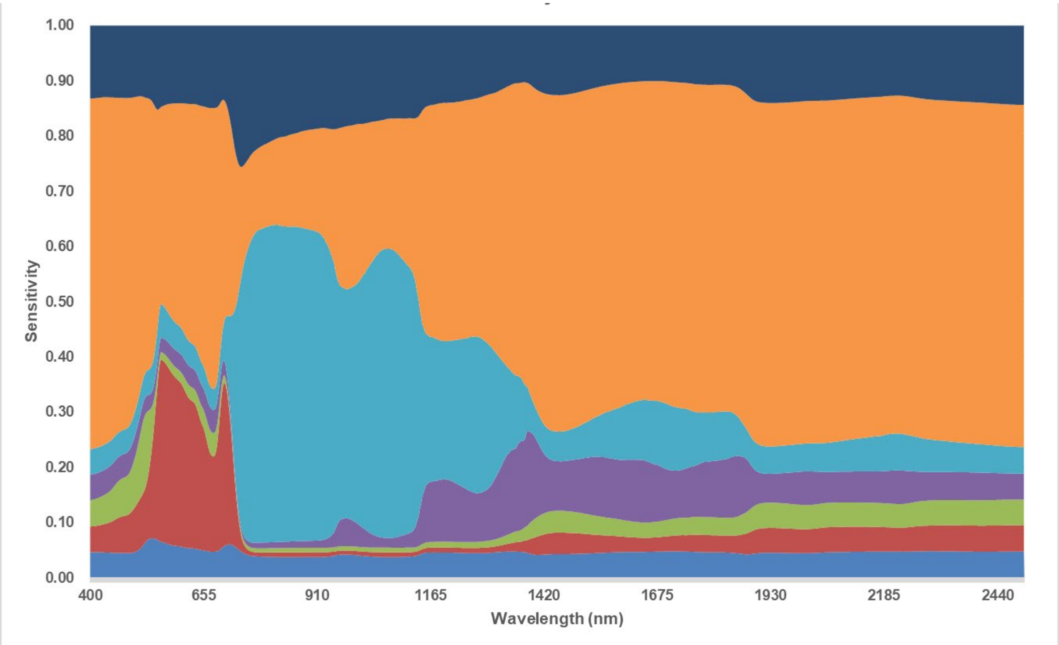

Supplement: Multimedia component 2 [file mmc2.pdf]
